# PARP1 depletion induces RIG-I-dependent signaling in human cancer cells

**DOI:** 10.1371/journal.pone.0194611

**Published:** 2018-03-28

**Authors:** Rajib Ghosh, Sanchita Roy, Sonia Franco

**Affiliations:** Department of Radiation Oncology and Molecular Radiation Sciences, Sidney Kimmel Comprehensive Cancer Center, Johns Hopkins University School of Medicine, Baltimore, MD, United States of America; University of St Andrews, UNITED KINGDOM

## Abstract

DNA Damage Response (DDR) and DNA repair pathways are emerging as potent, ubiquitous suppressors of innate immune signaling in human cells. Here, we show that human cells surviving depletion of the Single Strand Break (SSB) repair protein PARP1 undergo p21-dependent senescence or cell cycle checkpoint activation in the context of activation of innate immune signaling, or viral mimicry. Specifically, we observe induction of a large number of interferon-stimulated genes (ISGs) and multiple pattern recognition receptors (PRRs; including RIG-I, MDA-5, MAVS, TLR3 and STING) and increased nuclear IRF3 staining. Mechanistically, depletion of the double-stranded RNA (dsRNA) helicase RIG-I or its downstream effector MAVS specifically rescues ISG induction in PARP1-depleted cells, suggesting that the RIG-I/MAVS pathway is required for sustained ISG expression in this context. Experiments with conditioned media or a neutralizing antibody to the α/β-IFN receptor revealed that persistent ISG expression additionally requires an autocrine/paracrine loop. Finally, loss of PARP1 and radiation-induced DNA damage strongly synergize in the induction of p21 and ISGs. Overall, these findings increase our understanding of how PARP1 may suppress deleterious phenotypes associated to aging, inflammation and cancer in humans.

## Introduction

In response to viral infection, human cells activate an interferon (IFN)-dependent antiviral program [1]. Initially, specialized cytoplasmic sensors detect viral DNA or RNA and induce the expression of type I IFNs, including IFN-β and 13 IFN-αs [2, 3]. Secreted IFNs bind to their common IFN receptor, triggering JAK/STAT signaling and the coordinated induction of hundreds of interferon-inducible genes (ISGs)[4]. The products of these genes then execute the antiviral response via direct interaction with viral molecules as well as with many cellular factors that control cell cycle progression, apoptosis and other key cellular functions [1].

Type I IFN signaling can also be activated by cytoplasmic nucleic acids in the absence of viral infection (“viral mimicry”). In this context, DNA damaging agents are well-known to induce IFNs via multiple mechanisms (reviewed in [5]). For example, ionizing radiation (IR) triggers IFN signaling via accumulation of the pattern recognition receptor (PRR) cyclic GMP-AMP synthase (cGAS) at DNA breaks within radiation-induced micronuclei [6]. In addition, the dsRNA sensor RIG-I has also been widely implicated in the activation of viral mimicry after radiation or exposure to some chemotherapy agents [7], suggesting that both radiation-dependent RNA and DNA species may serve as substrates in this context. In addition, some drugs that alter the epigenetic landscape may also induce IFNs by de-repressing endogenous retroviruses [8, 9]. Indeed, both retroviral RNA and, upon reverse transcription, DNA are capable of triggering IFNs via specific PRRs [10].

In addition to exogenous treatments, intrinsic defects in DNA double-strand break (DSB) repair, commonly found in cancer cells and in normal cells with aging, are sufficient to promote innate immune signaling. For example, the DNA/cGAS/STING pathway is activated in cells deficient for the ATM kinase [11]. In addition to ATM, multiple factors involved in Homologous Recombination (HR), including BRCA1, BRCA2 and RAD51, function to suppress IFN signaling [12, 13], although the underlying mechanisms are incompletely understood [14]. In contrast to these studies on DSB repair proteins, roles for factors involved in Single-Strand Break (SSB) repair and other DNA repair pathways remain mostly unexplored.

PARP1, a ubiquitous SSB repair factor, is the founding member of a family of proteins with a carboxi-terminal PARP domain [15]. PARP1 binds to DNA breaks via its amino-terminal zinc fingers, resulting in a conformational change that activates its enzymatic activity [16]. PARP1-dependent poly(ADP-rybosyl)ation (PARylation) of histones and repair factors at the break site regulates their activity and ultimately promotes repair [17, 18]. In human and mouse cells, loss of PARP1 primarily results in a defect in the repair of Single-Strand Breaks (SSBs) [19, 20]. In dividing cells, this non-cytotoxic lesion is efficiently repaired via Homologous Recombination (HR) upon conversion to a DSB by the replication machinery [21, 22]. Consistent with these findings, mice and cells deficient for PARP1 do no show significant chromosomal instability unless challenged with SSB-inducing agents, such as ionizing radiation (IR) and alkylating agents [23]. In addition to its roles in DNA repair, PARP1 modulates other cellular processes, including transcription, metabolism and the response to various stresses [24].

To investigate how PARP1 maintains homeostasis in human cells, we have analyzed here the phenotypes of clonal populations emerging upon PARP1 gene inactivation via CRISPR/Cas9 editing of the PARP1 locus. We find that depletion of PARP1 triggers type I IFN signaling and identify the dsRNA sensor RIG-I and the type I IFN receptor as key elements in the maintenance of this IFN-dependent transcriptional program.

## Materials and methods

### Cells

HCT116 and HEK293T cells were grown in 10%FBS/DMEM supplemented with penicillin/streptomycin in a humidified incubator at 37°C. Both lines were authenticated using Short Tandem Repeat (STR) Profiling at the Johns Hopkins Genetics Resources Core Facility using the GenePrint 10 kit (Promega).

### Cloning and transfection of CRISPR/Cas9 constructs

Vectors pSpCas9(BB)-2A-Puro (PX459, expressing the Cas9 nuclease) and pSpCas9n(BB)-2A-Puro (PX462, expressing Cas9^D10A^ nickase) were obtained from Addgene [25]. For cloning, 2 μL of each oligonucleotide (100 μM) were diluted in 18 μL of 1X T4 ligase buffer, denatured at 95°C for 5 minutes and allowed to reanneal overnight. The oligonucleotides sequences were: exon 2, guide A: F: CACCGCCACCTCAACGTCAGGGTGC; R: AAACGCACCCTGACGTTGAGGTGGC; exon 2 guide B: F: CACCGTGGGTTCTCTGAGCTTCGGT; R: AAACACCGAAGCTCAGAGAACCCAC. For cloning of annealed oligonucleotides into PX459 or PX462, a simplified digestion/ligation reaction was performed. A 20 μL reaction containing 2 μL of annealed oligos (0.5 μM), 100 ng of vector, 1μL of FastDigest BbsI (Thermo Scientific) and 1μL of T4 ligase (New England Biolabs) was incubated in 1X FastDigest buffer at 37°C for 2 hours in a waterbath and used for bacterial transformation. To confirm the inserts, colony miniprep DNA was sequenced using a sequencing forward primer 5’-TGTAAACACAAAGATATTAG-3’. Plasmids were transfected into HEK293T cells using Fugene and HCT116 cells with Lipofectamine 3000 transfection reagents. For experiments aimed at testing the efficiency of editing, transfected cells were selected in puromycin (HCT116 = 1.5 ug/mL; HEK293T = 1 ug/mL) for 3–4 days and harvested for analyses by T7 assays and sequencing, as described below. For experiments aimed at generating KO lines, cells were subjected to sequential rounds of transfection/selection, cells were kept in conditions of exponential growth after the first selection and retransfected when puromycin sensitivity was restored. Cells were single cell subcloned in 96-well plates and expanded to 6-well plates for clone analysis.

### T7 Endonuclease I assay

After puromycin selection, genomic DNA was obtained using a standard phenol:chloroform extraction protocol. The target regions within exons 2 or 3 of PARP1 were PCR amplified with the following primers: exon2-F:; exon 2-R: exon3-F, exon 3-R. A 20-μL reaction containing 400 ng of PCR product in 1X NEB buffer 2 was subjected to denaturation/reannealing in a thermocycler (95°C, 5 min; 95–85°C at −2°C/s; 85–25°C at −0.1°C/s) to allow for annealing of mismatched DNA molecules. After addition of 1 μL of T7 endonuclease (New England Biolabs, MO302), samples were incubated at 37°C for 1 hour, resolved in a 2% agarose gel and photograph using a GelDoc apparatus (BioRad).

### TOPO-TA cloning and sequencing

The targeted regions were amplified from gDNA as above and cloned into TOPO-TA (ThermoFisher Scientific) following manufacturer instructions. Colony miniprep DNA was sequenced at the Johns Hopkins Genetics Core Facility and aligned to the consensus PARP1 sequence.

### Proliferation assay

For HEK293T cells, we seeded 10^5^ cells in 10-cm dishes and, after 10 days, cells were counted using a Celometer Auto T4 cell counter. For HCT116 cells, 7.5 X 10^4^ cells were plated on each well of 6 well plates in triplicate and counted after each day for 2–3 days.

### Clonogenic assay

We seeded 200 cells in each well of 6-well plates. After 15 days, colonies were stained with crystal violet and counted. Representative images were taken using a digital camera.

### Murine xenograft experiments

HCT116^EV^ and HCT116^*PARP1*-/-^ cells (1x10^7^) were mixed with Growth Factor Reduced Matrigel (Corning, Cat # 356230) 1:1 and injected into the flanks of NSG mice. Tumor diameters were measured using a caliper and tumor volume was calculated using the formula (L*W^2^)/2.

### Cell cycle analysis

To quantify cell cycle distribution, exponentially growing cells were fixed in cold 70% ethanol, permeabilized in Triton-X, digested with RNAse A and stained with propidium ioidide (PI). All data was acquired using a FACSCalibur and analyzed with FlowJo software.

### Staining for senescence-associated β-galactosidase (SA β–gal)

Cells were fixed and incubated in β–gal using a commercial kit (Cell Signaling Technology, #9860), following the manufacturer’s instructions. For quantification, images were taken using an EVOS microscope and we quantified n = cells per field; n = 2 fields per well in triplicate wells of a 6-well plate.

### RNA-Seq and data analysis

Total RNAs were isolated from cells in culture using RNAeasy mini kit and on column DNase I treatment following manufacturers instruction (Qiagen, Germantown, MD). One microgram of total RNA from each sample were submitted to the Johns Hopkins University Deep Sequencing and Microarray Core for sequencing and analyses. Briefly, libraries are prepared using Illumina TruSeq stranded total RNA sample preparation kit following the manufacturer's recommended procedure. All six samples are pooled together and sequenced on Illumina NextSeq using a 300cycle High Output reagent kit for paired end 100bp sequencing. The illumina NextSeq RTA software version 2.4.11 was used for base calling and illumina bcl2fastq software version 2.17.1.14 was used to generate fastq files and trimming adapter sequences. RNAseq data is aligned to human reference genome hg19 using tophat 2 (version 2.0.14) and differential expression detected using cufflink software (version 2.2.1). This final differentially expressed gene table with their respective absolute expression values were used to run Ingenuity Pathway Analyses (IPA, Qiagen), Gene Set Enrichemnt assay (GSEA, Broad Institute, MIT), Gene Ontology on the Panther Gene ontology platform[26], and Cufflinks [27, 28].

### Quantitative RT-PCR (q-RT-PCR)

Total RNA was reverse-transcribed into cDNA using SuperScript® III Reverse Transcriptase System (Thermo Fisher Scientific Inc. Waltham, MA) and oligo(dT)20 primers. All qPCR experiments were performed using ddCt method and PowerUp SYBR Green Master Mix (Applied Biosystems, Carlsbad, CA) on a CFX384 BioRad Real-Time PCR System (BioRad, Hercules, CA). 18S was used as the endogenous control. All primers used in this study are listed in the S7 Table.

### Staining for nuclear IRF3

Cells were fixed in 4% formaldehyde for 5 min followed by cold methanol 10 min and stained with a rabbit polyclonal antibody to IRF3 (clone SL12; Santa Cruz; sc-33641). After incubation with a secondary antibody (Cy3-conjugated goat anti-mouse IgG (115-166-071, Jackson ImmunoResearch), cells were mounted in Vectashield with DAPI. Images were acquired using a Zeiss Axioplan Imager Z.1 microscope equipped with a Zeiss AxioCam and an HXP120 mercury lamp (Jena GbH) and analyzed using dedicated software (Zeiss Axiovision Rel4.6). For comparison, we recorded five random fields using a 60X objective within each sample.

### Immunoblotting

Cells were resuspended in RIPA buffer supplemented with PMSF, protease inhibitors and the phosphatase inhibitors sodium fluoride, sodium orthovanadate and β-glycerophosphate. Fifty μg of protein were resolved via SDS-PAGE, transferred to PVDF membranes and blotted with antibodies to the following proteins: PARP1 (clone C2-10; Trevigen, #4338-MC-50); p53 (clone 1C12; Cell Signaling); phospho-p53 (Ser15) (Cell Signaling); p21 (clone 12D1, Cell Signaling); PAR (4335-MC; Trevigen), γ-H2AX (Millipore; #05–636), phospho-KAP1 (Ser824) (Bethyl Laboratories, A300-767A); phospho-CHK2 (Thr68; Cell Signaling, #2661); STING (MAB7169, R&D Systems); MDA-5 (Cell Signaling Technology; #5321); MAVS (Cell Signaling Technology; #3993); RIG-I (clone D14G6; Cell Signaling Technology; #3743); TLR3 (clone D10F10; Cell Signaling Technology; #6961). Secondary antibodies were HRP-linked anti-mouse (Cell Signaling; 7076) and HRP-linked anti-rabbit IgG (Cell Signaling; 7074). To control for loading, blots were hybridized with an HRP-conjugated antibody to GAPDH (clone 14C10; Cell Signaling, #3683S).

### RNA silencing

Cells (2x10^5^ per 6-cm dish) were transfected with siRNAs (using the Lipofectamine RNAiMax (Lifetechnologies, Carlsban, CA) following the manufacturer’s protocol. Cells were harvested for analysis 96 hrs post transfection. All siRNAs were purchased as SMARTpools from Dharmacon and used at 100 nM. Specifically, we used products L-012511-00-0005 (DDX58/RIG-I); L-013041-00-0005 (IFIH1/MDA-5); L-024237-00-0005 (MAVS); L-024333-02-0005 (TMEM173/STING) and L-007745-00-0005 (TLR3). As a control, we transfected cells with a pool of nontargeting siRNAs (ON-TARGETplus Nontargeting Pool; Dharmacon Cat# D-001810-10).

### Conditioned media experiments

HCT116^EV^ and HEK293T^EV^ cells (4x10^5^ cells) were seeded in 6-cm dishes with conditioned media from either EV cells or PARP1 KO clones for each line. Conditioned media was replaced once after 2 days and cells were harvested and counted at day 5.

### Blocking of the α/β-IFN receptor

Cells (2.5 x 10^4^ cells per well in a 6-well plate) were incubated with anti-human interferon α/β receptor chain 2 (IFNR) antibody, clone MMHAR-2 (PBL Interferon Source, 21385–1) or with an isotype control (Purified mouse IgG2a, kappa isotype, clone MG2a-53, Biolegend, #401501) and counted or harvested for RNA after 72 hours.

### Irradiation

Cells were either mock-irradiated or irradiated using a CIXD X-Ray irradiator (dual x-ray tube system), Xstrahl Ltd., UK, operating at a dose rate of 3.93 Gy/min.

### Drug treatments

Methylmethane sulfonate (MMS) was purchased from Sigma (#129925). Olaparib/AZD2281 was purchased from Selleckchem (#S1060). For experiments aimed at reverse transcriptase inhibition, didanosine (S1702), neviparine (S1742) and zidovudine (S2579) were all purchased from Selleckchem. Cells (2x10^5^ cells per 6-cm dish) were incubated with all three compounds or vehicle for 72 hours prior to harvesting for RNA.

### Transient transfection of PARP1 cDNA

Cells (2x10^5^ per 6-cm dish) were transfected with pEGFP-hPARP1-C1 plasmid using Lipofectamine 3000 (Invitrogen, Carlsbad, CA) and harvested 72 hours post transfection for cell counts and RNA.

### Lentiviral infection for PARP1 reconstitution

Lentiviral vectors pEX-C0028-Lv105, expressing PARP1, and pReceiver-Lv105 control vector were purchased from Genecopeia, Rockville, MD. Target cells were infected with viral supernatants overnight twice in the presence of 8 μg/ml polybrene (Sigma Aldrich, St. Louis, MO).

### Quantification of telomere length by FISH on metaphases

Cells were incubated in 0.1 μg/mL colcemid (KaryoMAX, Gibco) for 4 hr, swollen in 0.45% KCl for 30 min at 37°C and fixed in methanol/acetic acid (3/1). Metaphases were hybridized with a telomere peptic nucleic acid (PNA) probe as described [29]. Briefly, slides were fixed in 4% formaldehyde and digested in pepsin prior to denaturation at 80°C for 3 min. Slides were then hybridized with a Cy3-labeled (TTAGGG)_3_ PNA probe (Applied Biosystems), washed and mounted in Vectashield with DAPI (Vector Laboratories, Burlingame, California). Images were obtained using a Zeiss Axioplan Imager Z.1 microscope equipped with a Zeiss AxioCam and an HXP120 mercury lamp (Jena GmbH) and dedicated software (Zeiss Axiovision Rel 4.6). For quantitative analysis of telomere length on metaphases, we employed TFL-Telo software (kind gift of Dr. Peter Lansdorp). We analyzed 10 metaphases per sample.

### Statistical analysis

Data is represented as the mean and standard deviation (s.d.) of at least 3 replicates within each experiment. For two group comparisons, an unpaired two-tailed Student’s *t*-test was performed using Excel software to calculate the statistical significance of the difference in mean values and the *P* value. A *P* value of <0.05 was considered statistically significant. *P<0.05; **P<0.01; ***P<0.001.

## Results

### Acute PARP1 depletion triggers a stable pro-survival program that limits cell proliferation

**Fig 1:**
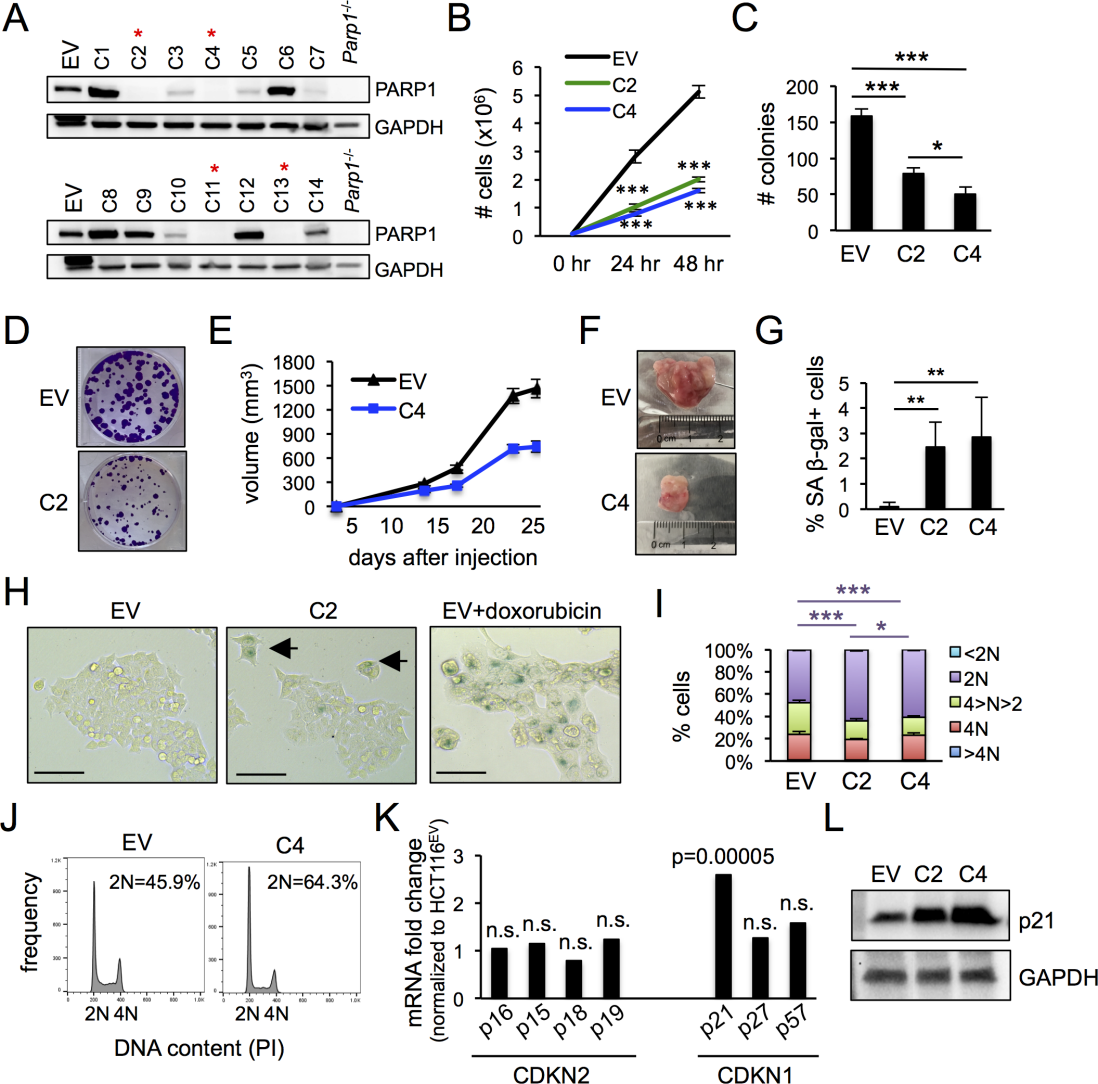
HCT116^*PARP1*-/-^ cells show defects in proliferation and increased senescence. (A) After editing PARP1 exon 2 via CRISPR/Cas9 ^D10A^-dependent “double nicking”, single cell-derived subclones were screened by immunoblotting. Red asterisks mark PARP1 “knock out” (KO) clones. EV, extracts from HCT-116 cells transfected with an “empty vector” plasmid expressing Cas9^D10A^ but no gRNAs. Extracts from *Parp1*^-/-^ B cells were included as a negative control. (B) To quantify proliferation, 10^5^ cells were seeded in 60 mm plates and counted at 24 and 48 hours. Data represents the mean and standard deviation of 3 independent experiments. (C-D) Clonogenic assay. Bars in (C) represent the average and standard deviation of 3 samples in 3 independent experiments. Representative examples are shown in (D). (E-F) Xenograft assay. HCT116^EV^ and HCT116^PARP1-/-^ cells (clone C4) were injected into NGS mice and tumors were measured at the indicated timepoints. Each data point in (E) represents the average and standard error of the mean (s.e.m.) of 10 tumors per line. Representative tumors at day 25 are shown in (F). (G-H) Cells were stained with senescence-associated β-galactosidase pH = 6.0 (SA β-gal) and the percentage of positive cells was quantified using brightfield microscopy. Data in (G) represents the average and standard deviation of 6 wells per cell line, N = 500 cells/well, pooled from two independent experiments. Representative examples are shown in (H). Black arrows point to senescent cells. As a positive technical control, HCT116^EV^ cells were treated with 10μM doxorubicin for 2 hours to induce senescence. (I-J) Cell cycle analysis after staining with propidium iodide (PI). Bars in (I) indicate the average and standard deviation of 3 independent experiments. Representative examples of cell cycle distributions are shown in (J). (K) Analyses of RNA-Seq data for members of the CDKN2 and CDKN1 families of cell cycle regulators. The fold change and p value were generated by comparing the expression of two KO clone to the EV control, as described in the Methods section. (L) Cell extracts were probed with antibodies to p21 and, as a loading control, GAPDH.

To “knock out” (KO) PARP1 in human cells, we introduced DSBs at PARP1 exon 2 using a pair of gRNAs coupled to Cas9^D10A^ (“double nicking” [30]). When tested in HEK293T cells, this approach resulted in frequent deletions and indels at the target locus, as determined via the T7 assay and Sanger sequencing (S1 Fig). Moreover, editing of the target sequence was comparable after “double nicking” or expression of individual gRNAs (A or B) with the Cas9 nuclease, while co-expression of gRNAs A or B with Cas9^D10A^ did not result in measurable editing (S1 Fig). These data indicate that “double nicking” of the PARP1 locus is highly efficient and specific, as previously described for other loci [30]. Using these reagents, we inactivated the two PARP1 alleles in HCT116 cells, a colon cancer cell line with a stable, diploid karyotype and functional p53 [31]. Consistent with efficient editing of the PARP1 locus in this line (S2 Fig for T7 assay), 4/14 (28.5%) single cell-derived clones lacked PARP1 expression (Fig 1A).

We selected two HCT116^*PARP1*-/-^ clones, C2 and C4, for additional studies. Sequencing of the target region revealed deletions and indels predicted to result in truncations and null alleles and confirmed their clonal origin from independent events (S2 Fig). The proliferation rate of both clones was markedly reduced relative to control cells that had been transfected with an empty vector expressing Cas9^D10A^ but no gRNAs (HCT116^EV^ cells; Fig 1B). Similarly, cells surviving PARP1 depletion showed reduced clonogenic capacity (Fig 1C and 1D) and slower growth in a xenograft assay (Fig 1E and 1F). Staining for the senescence marker senescence-associated β-galactosidase pH = 6.0 (SA β-gal) revealed that loss of PARP1 resulted in a statistically significant increase in the frequency of senescent cells (from less than 0.1% in control PARP1-proficient cultures to 2.4 and 2.8% in C2 and C4 cultures, respectively; Fig 1G and 1H). As expected, SA β-gal-positive cells showed characteristic morphological changes, including larger size and multinucleation (Fig 1H). In addition, cycling cells in PARP1-deficient cultures also progressed slower through the cell cycle due to a marked accumulation of cells with 2N DNA content (Fig 1I and 1J). Indeed, RNA-Seq analysis of the two PARP1-deficient clones relative to control HCT116^EV^ cells revealed selective induction of p21, a main regulator of the G1/S checkpoint, in PARP1-deficient cells (Fig 1K; S1 Table for global RNA-Seq data). Consistent with mRNA induction, the expression of protein was also markedly increased and inversely correlated with the proliferation rate (higher expression of p21 in clone C4 with more marked proliferative defect; Fig 1L).

To further examine the generality of these findings, we employed the same reagents and approaches to inactivate PARP1 in a nonepithelial cell line, HEK293T cells. Unlike HCT116 cells, HEK293T cells are characterized by an unstable karyotype and loss of p53 function due to the expression of SV40 large T. Screening of 16 HEK293T clones obtained from cells that survived editing revealed 6 (37.5%) PARP1 KO clones (HEK293T^*PARP1*-/-^; S3 Fig). Similar to our observations above in HCT116 cells, all PARP1-deficient clones showed reduced proliferative rate, clonogenic potential and growth in a xenograft assay (S3 Fig). Moreover, HEK293T^*PARP1*-/-^ cultures also showed increased frequency of senescent cells and accumulation of cells with 4N DNA content, correlating with increased p21 mRNA and protein (S3 Fig). Taken together, these findings reveal that survival after PARP1 loss relies on an adaptive program that suppresses cell death at the expense of slower cell cycle progression and increased senescence.

### PARP1 reconstitution partially rescues phenotypes in cells surviving PARP1 depletion

To examine whether the adaptive changes described above can be reverse by restoring PARP1 expression, we next attempted to generate stable, PARP1-reconstituted HCT116 ^*PARP1*-/-^ and HEK293T^*PARP1*-/-^ cells via infection with lentiviral vectors harboring the PARP1 cDNA. However, we failed to recover PARP1-proficient HCT116 cells, consistent with previous reports of defective lentiviral integration in PARP1-deficient cells [32]. However, we were able to propagate a fraction of surviving HEK293T^*PARP1*-/-^ cells as polyclonal, stable lines. The level of PARP1 in these lines was comparable to (clone C9) or lower than (clone C5) the endogenous level (S4 Fig). Moreover, the reconstituted protein was active, as determined via a PARylation assay and its ability to rescue PARP inhibitor resistance characteristic of PARP1-deficient cells [33] (S4 Fig). However, PARP1 re-expression did not significantly rescue phenotypes of cell proliferation and cell cycle distribution in the reconstituted cells (S4 Fig). Similarly, transient expression of PARP1 in HCT116^*PARP1*-/-^ cells at a level comparable to endogenous levels partially rescued olaparib resistance and baseline proliferative defects (S5 Fig). Altogether, these findings suggest that PARP1 reconstitution partially rescues phenotypes of growth and olaparib resistance in PARP1-deficient cells.

### Cells surviving PARP1 depletion are hypersensitive to SSB-inducing agents

**Fig 2:**
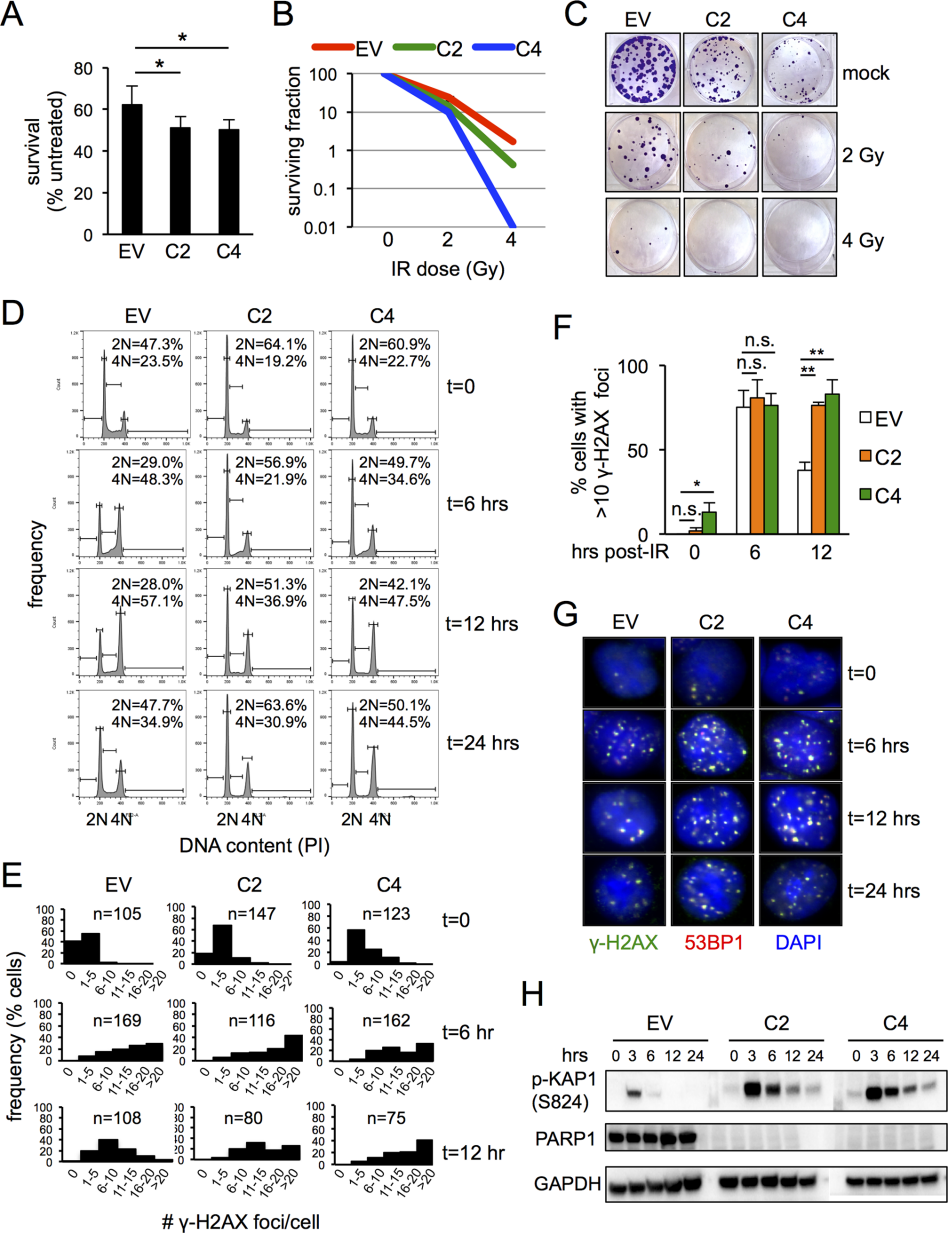
HCT116^*PARP1*-/-^ cells are radiosensitive. (A) HCT116^*PARP1*-/-^ cells (clones C2 and C4) and control PARP1-proficient HCT116^EV^ cells were exposed to IR (5 Gy) and counted after 48 hours. Bars represent the average and standard deviation of triplicates. Data is representative of two independent experiments. (B-C) Clonogenic assay after exposure to the indicated doses of IR. The surviving fraction is plotted in (B) and representative plates are shown in (C). Data is representative of two independent experiments. (D) Cell cycle profile after IR (5 Gy) at the indicated timepoints (hours after radiation). The percentage of cells with 2N and 4N DNA content is indicated. Data is representative of three independent experiments. (E-G) Quantification of irradiation-induced foci (IRIF). Cells were exposed to IR (2 Gy) and the number of foci per nucleus quantified by indirect immunofluorescence with antibodies to γ -H2AX and 53BP1. Histograms on (E) show the distribution of γ-H2AX foci per nucleus at baseline and 6 and 12 hours after IR. The percentage of cells with more than 10 γ-H2AX foci at the same timepoints is shown in F. Bars represent the average and standard deviation of three fields, N = 100 cells/field. (H) Cells extracts were harvested at the indicated timepoints after IR (4 Gy) and probed with antibodies to phospho-KAP1 (S824) and, as a loading control, GAPDH.

PARP1 is required for SSB repair and PARP1-deficient cells accumulate DSBs upon replication of SSB. In contrast, PARP1 is dispensable for DSB repair *per se* via either Homologous Recombination (HR) or canonical NonHomologous End Joining (NHEJ). To characterize DNA repair in HCT116^*PARP1*-/-^ cells, we assessed the kinetics of cell cycle progression and DDR activation in response to IR, an agent that introduces SSBs and, to a lesser extent, DSBs (Fig 2). Relative to PARP1-proficient controls, HCT116^*PARP1*-/-^ cells showed decreased proliferation (Fig 2A) and clonogenic capacity (Fig 2B and 2C) associated to persistent activation of radiation-induced cell cycle checkpoints (Fig 2D), delayed resolution of IR-induced γ-H2AX foci (Fig 2E–2G) and persistent expression of phospho-KAP1 (Ser824) (Fig 2H), a marker of ATM activation. These phenotypes likely represent increased generation of DSBs upon replication of IR-dependent SSBs rather than a DSB repair defect *per se*. In support of this notion, telomere-FISH on HCT116^*PARP1*-/-^ metaphase spreads failed to reveal chromosomal breaks (N = 20 and N = 19 metaphases for clones C2 and C4, respectively; see S6 Fig for representative examples). Moreover, HCT116^*PARP1*-/-^ cells were also hypersensitive to MMS, an alkylating agent that primarily introduces SSBs (S7 Fig). Finally, HEK293T^*PARP1*-/-^ cells similarly showed persistent activation of cell cycle checkpoints and DDR markers γ-H2AX, phospho-KAP1 (Ser824) and phospho-CHK2 (Thr68) after IR (S8 Fig) and MMS hypersensitivity (S7 Fig).

### Cells surviving PARP1 depletion activate innate immune signaling

To gain further insights into the pathways that promote cell survival upon PARP1 loss, we analyzed global RNA-Seq data for each line (S1 Table for HCT116 data, S2 Table for HEK293T data and S9 Fig for volcano and scatter plots for both lines). As expected, direct comparison of differentially regulated mRNAs between the two cell lines showed little overlap (S9 Fig). In contrast, pathway analysis using Gene Ontology (GO) revealed significant functional overlap. In particular, enrichment for mRNAs related to binding/protein binding/receptor binding was prominent after PARP1 depletion in the two lines (S10 Fig), suggesting a role for autocrine/paracrine mechanisms in the observed phenotypes. Additional analysis using Gene Set Enrichment Analysis (GSEA) confirmed enrichment for common pathways (S3 Table and S4 Table for HCT116 and HEK293T cells, respectively). In particular, “Interferon Alpha Response”, “Interferon Gamma Response”, “Inflammatory Response” and “Complement” were differentially regulated in both lines, pointing to alterations in innate immune system in survivors. Similarly, Ingenuity Pathway Analysis (IPA) revealed enrichment for genes related to immune function (S5 Table and S6 Table for HCT116 and HEK293T cells, respectively).

**Fig 3:**
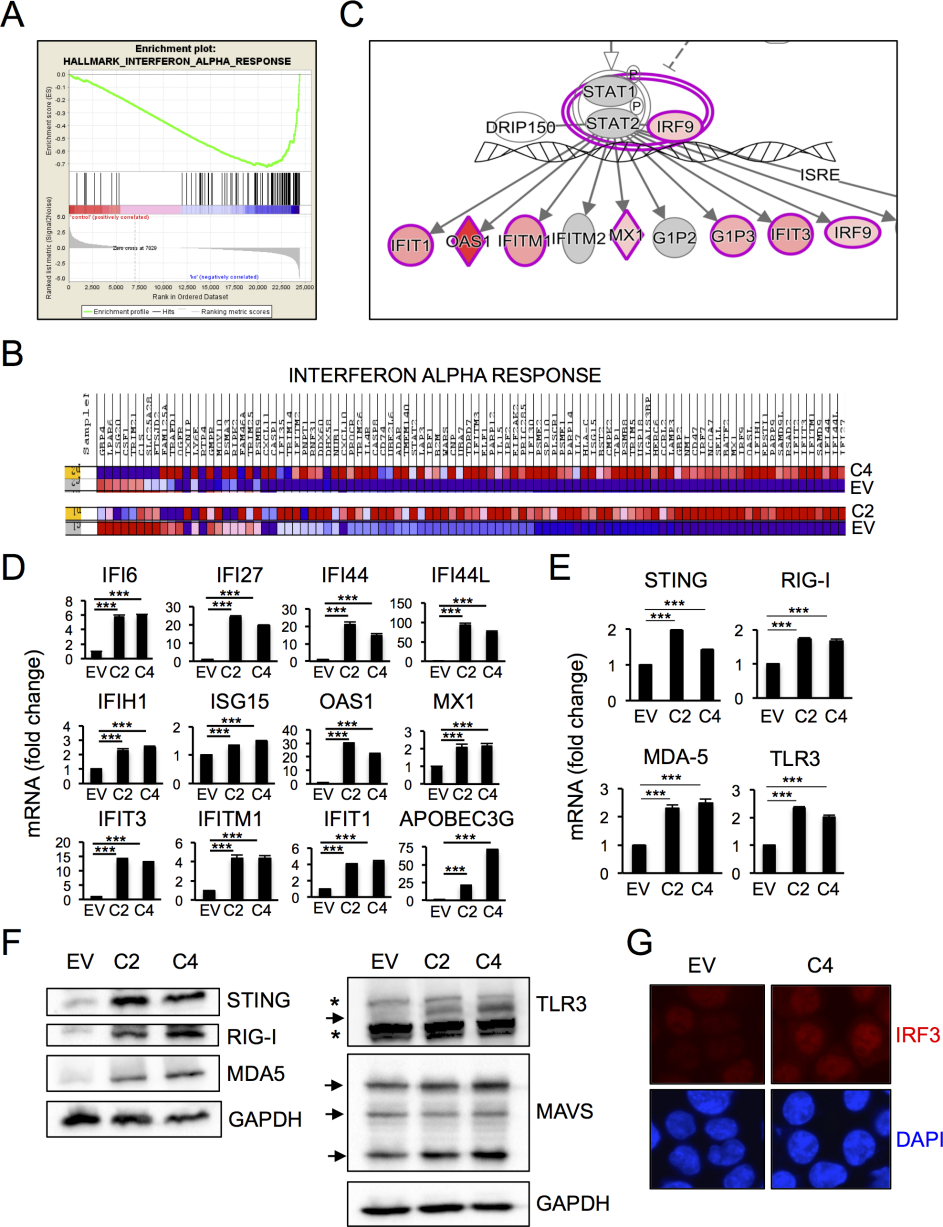
HCT116 cells surviving PARP1 depletion activate innate immune signaling. (A-B) RNA-Seq data from HCT116^EV^ and HCT116^*PARP1*-/-^ cells (clones C2 and C4) was analyzed using Gene Set Enrichment Analysis (GSEA). The top category of differentially expressed genes was “Interferon Alpha Response”. The enrichment plot is shown in (A) and the heat map for the 97 mRNAs in this category in shown in (B). Map was generated using Cufflinks software (version 2.2.1) and shows absolute expression values independently normalized and analyzed for each comparison pair (EV/C2 and EV/C4). (C) The same RNA-Seq dataset was analyzed using Ingenuity Pathway Analysis (IPA). A representative plot highlighting enrichment for Interferon-Stimulated Genes (ISGs) is shown. (D) The induction of multiple ISGs observed by RNA-Seq was confirmed by q-RT-PCR. Bars represent the average and standard deviation of quadruplicate samples in each experiment and data is representative of 2–4 independent experiments. (E-F) The induction of factors involved in the sensing/signaling of cytoplasmic nucleic acids observed by RNA-Seq was confirmed by Q-RT-PCR (E). Bars represent the average and standard deviation of quadruplicate samples in each experiment and data is representative of 2–3 independent experiments. Protein expression for the same factors was assessed by immunoblotting (F). Blots are representative of 2–3 independent experiments. (G) Fixed cells were stained with an antibody to IRF3 and counterstained with DAPI. Images are representative of 5 random fields per slide. The experiment was repeated twice with similar results.

In addition, these analyses revealed enrichment for cell line-specific pathways. Of note, the mRNA dataset for HCT116^*PARP1*-/-^ cells was highly enriched for signatures related to “viral mimicry”, such as “Interferon Alpha Response” (top one category in GSEA analysis; Fig 3A and 3B); “Role of Pattern Recognition Receptors in Recognition of Bacteria and Viruses” and “Communication Between Innate and Adaptive Immune Cells”. Consistent with the GSEA analyses, IPA revealed enrichment for mRNAs downstream of the α/β-interferon receptor, including many interferon-stimulated genes (ISGs; Fig 3C). Using qRT-PCRs, we validated the induction of a large subset of ISGs in HC116A^*PARP1*-/-^ cells, including IFI6, IFI27, IFI44, IFI44L, IFIT1, IFIT3, IFITM1, IFITH1, ISG15, OAS1, MX1 and APOBEC3G (Fig 3D).

In addition, analyses of RNA-Seq data revealed induction for several factor that function to sense and signal cytoplasmic DNA (STING) or RNA (RIG-I, MDA-5 and TLR3) during viral mimicry (S1 Table). These findings were confirmed by q-RT-PCR (Fig 3E). Interestingly, although these factors lack an interferon-stimulated response element (ISRE) and were induced modestly (1.5–2.0 fold), the increase in protein level was dramatic (Fig 3F). Consistent with previous findings by Barber and colleagues [34], we failed to detect cGAS mRNA in control HCT116^EV^ cells (S1 Table). Moreover, cells surviving PARP1 similarly fail to express cGAS mRNA (S1 Table), suggesting that they maintain epigenetic repression of the cGAS locus [34]. Finally, we also observed increased nuclear translocation of the transcription factors IRF3 (Fig 3G), a main downstream effector of both cytoplasmic DNA and RNA signaling pathways.

In contrast, viral mimicry was less prominent in HEK293T^*PARP1*-/-^ survivors. Although GSEA analysis also revealed enrichment for “Interferon Alpha Response” in HEK293T cells (S4 Table and S11 Fig), it was less marked than for HCT116 cells (ES, -0.58 and -0.72 for HEK293T and HCT116 cells, respectively). Consistent with these findings, mRNAs of 12 ISGs highly induced in HCT116^*PARP1*-/-^ cells were variably induced in their HEK293T^*PARP1*-/-^ counterparts (S11 Fig). The mRNAs for 4 ISGs (OAS1, IFIT3, MX1 and IFITH1) were induced in both clones, but the fold change tended to be lower than for HCT116 cells. The remaining 8 ISGs were induced in only one of the two clones, not altered or even repressed (S11 Fig). These differences between the two lines were not due to clear differences in the baseline levels of ISG expression in the parental cells (S12 Fig). Moreover, although we clearly observed induction of MDA5, RIG-I, STING and, to a lesser extent, MAVS in HEK293T^PARP1-/-^ cells relative to HEK293T^EV^ cells, the extent of induction was also generally lower than that observed in HCT116 cells examined in parallel (see S13 Fig for side by side comparison of the two lines). We did not detect TLR3 expression in either HEK293T^EV^ or HEK293T^*PARP1*-/-^ cells (S13 Fig), consistent with their previously described TLR^low^ phenotype [35]. Based on these findings, experiments below focus on HCT116^*PARP1*-/-^ cells as a model to further investigate the regulation of viral mimicry in PARP1-depleted cells.

### ISG induction in PARP1-depleted cells is dependent on the RIG-I RNA helicase and its downstream effector MAVS

**Fig 4:**
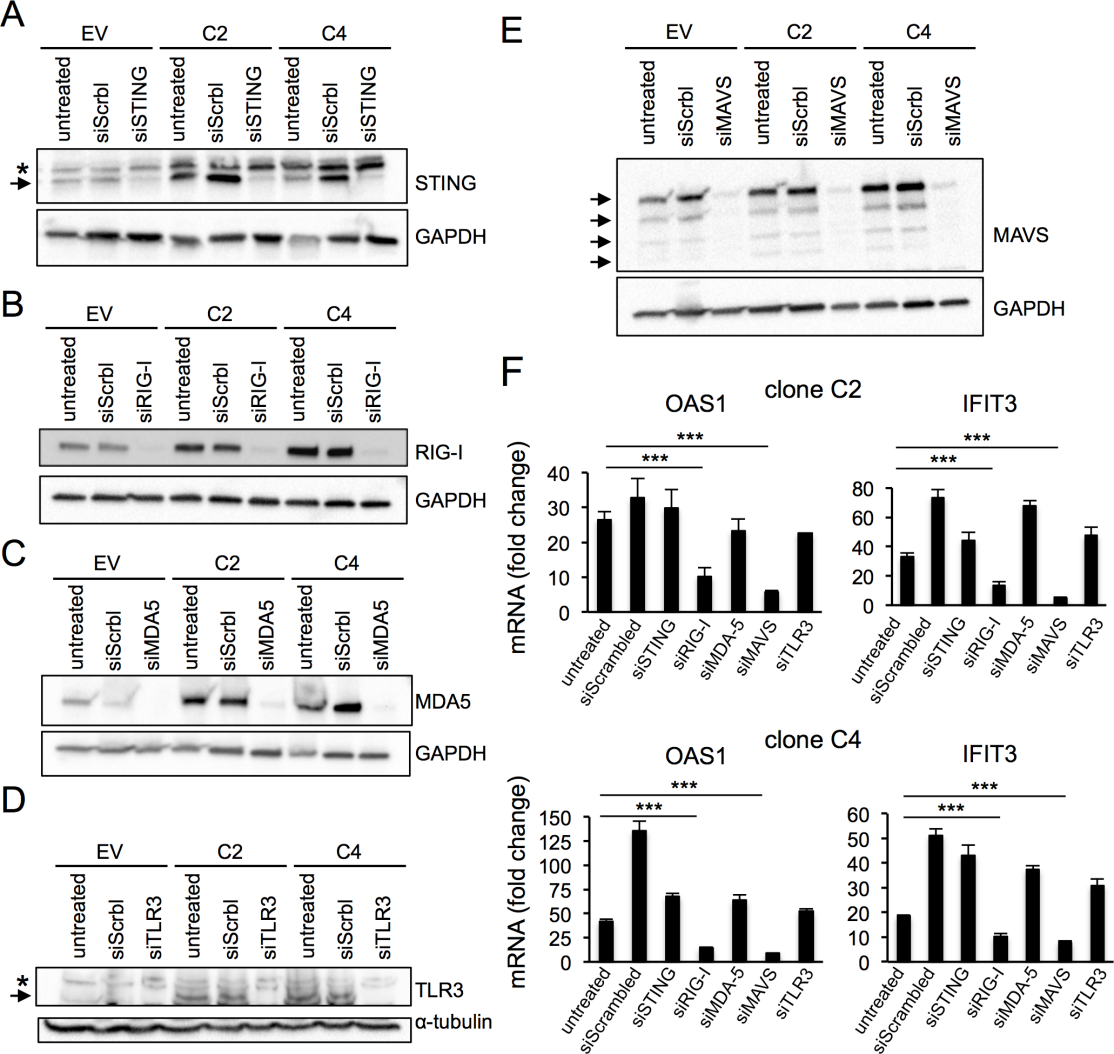
ISG induction in cells surviving PARP1 depletion is dependent on RIG-I and MAVS. (A-E) To assess the efficiency of RNA silencing to STING (A), RIG-I (B), MDA-5 (C), TLR3 (D) or MAVS (E), cells transfected with the specific pooled siRNAs and control cells transfected with a scrambled siRNA or untreated were harvested 4 days after transfection and probed with the indicated antibodies. GAPDH (A, B, C, E) or α-tubulin (D) served as loading controls. In blots where multiple bands are observed (A, D, E), black arrows point to specific bands and asterisks to unspecific bands. (F) The expression of ISGs OAS1 and IFIT3 was quantified by qRT-PCR after knock down. Data is normalized to the expression in HCT116^EV^ cells (= 1). Bars represent the average and standard deviation of quadruplicates. Data is representative of two independent experiments.

Viral mimicry is triggered by the detection of cytoplasmic nuclei acid species by specialized pathways [36]. Interestingly, we find that factors involved in the detection/signaling of both DNA (STING) and RNA (RIG-I, MDA5, TLR3 and MAVS) accumulated in cells surviving PARP1 depletion (Fig 3E and 3F above). To address the functional significance of their induction, we next depleted HCT116^*PARP1*-/-^ cells of each factor using pooled siRNAs and assessed its effect on the expression of two reporter ISGs, OAS1 and IFIT3 (Fig 4). For each factor, knock down was highly efficient, with typically less than 10% residual mRNA at day 4 after transfection (S14 Fig). Efficient protein depletion was confirmed by immunoblotting (Fig 4A–4E). Interestingly, depletion of the RNA sensor RIG-I or its downstream effector MAVS markedly reduced the expression of both ISGs in the same experiments (Fig 4F). This effect was specific, because depletion of MDA-5, which also functions in the recognition of cytoplasmic dsRNA and signals via MAVS, had no effect (Fig 4F). Depletion of STING had no effect on ISG expression (Fig 4F), although the lack of cGAS expression in HCT116 cells [34] complicates the interpretation of this finding (see Discussion below). Finally, depletion of TLR3, that senses dsRNA in the endosomal compartment, had no effect of ISG induction (Fig 4F). We conclude that the RIG-I/MAVS pathway is necessary to sustain ISG expression in HCT116 cells surviving PARP1 depletion.

### Constitutive ISG induction in cells surviving PARP1 depletion is maintained via an autocrine/paracrine loop involving the interferon receptor

**Fig 5:**
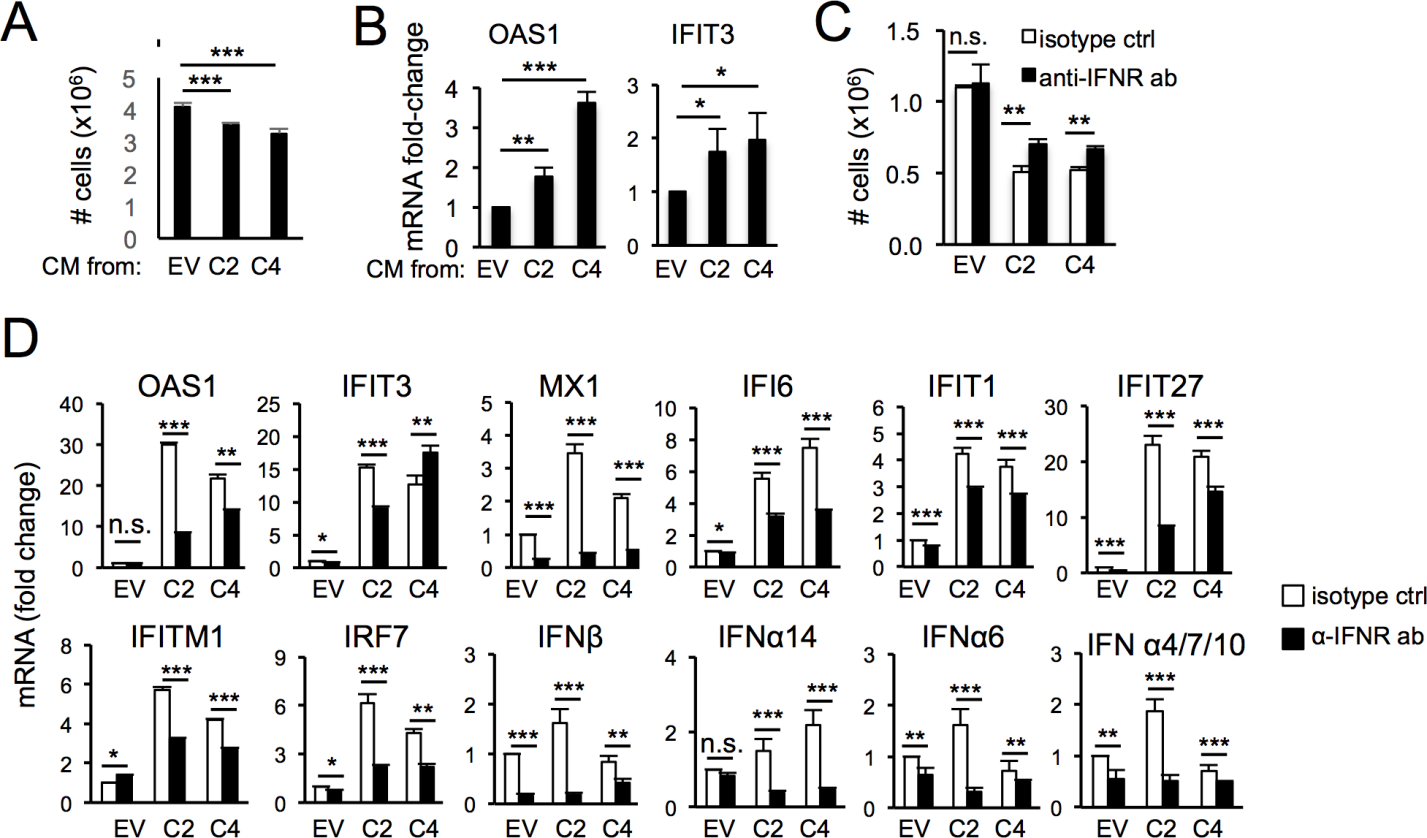
ISG induction in cells surviving PARP1 depletion requires secreted factors and the α/β-IFN receptor. (A-B) HCT116^EV^ were incubated with conditioned media from either HCT116^EV^ or HCT116^*PARP1*-/-^ cells (clones C2 and C4) for 48 hours and the number of cells counted in triplicates (A). In parallel, OAS1 and IFIT3 mRNAs were quantified by q-RT-PCR (B). Data is normalized to the expression in cells incubated with conditioned media from HCT116^EV^ cells. (C-D) HCT116^EV^ and HCT116^*PARP1*-/-^ cells (clones C2 and C4) were incubated with an antibody to the α/β-IFN receptor or an isotype control for 96 hours and the number of cells counted in triplicates (C). For the same samples, mRNAs for β-IFN, several α-IFNs and the indicated ISGs were quantified by qRT-PCR (D). Data is normalized to expression in HCT116^EV^ cells treated with an isotype control. Data is representative of two independent experiments.

Upon IFN induction downstream from cytoplasmic sensors, secreted IFNs bind to their receptors to maintain and amplify ISG expression via JAK/STAT signaling [1]. To assess whether this pathway mediates ISG induction in PARP1-depleted cells, we next determined whether conditioned media from PARP1-deficient cells could influence IFN signaling in PARP1-proficient target cells. Addition of conditioned media from HCT116 ^*PARP1*-/-^ cells to HCT116^EV^ cells induced growth arrest (Fig 5A) and ISG mRNA expression (Fig 5B). Importantly, blocking of the α/β-IFN receptor partially rescued growth arrest in HCT116 ^*PARP1*-/-^ cells (Fig 5C) and markedly reduced the expression of β-IFN, several α-IFNs and many ISGs (Fig 5D), suggesting that secreted type I IFNs may mediate this effect, at least in part.

### Interferon signaling modulates radiation response in HCT116^*PARP1*-/-^ cells

**Fig 6:**
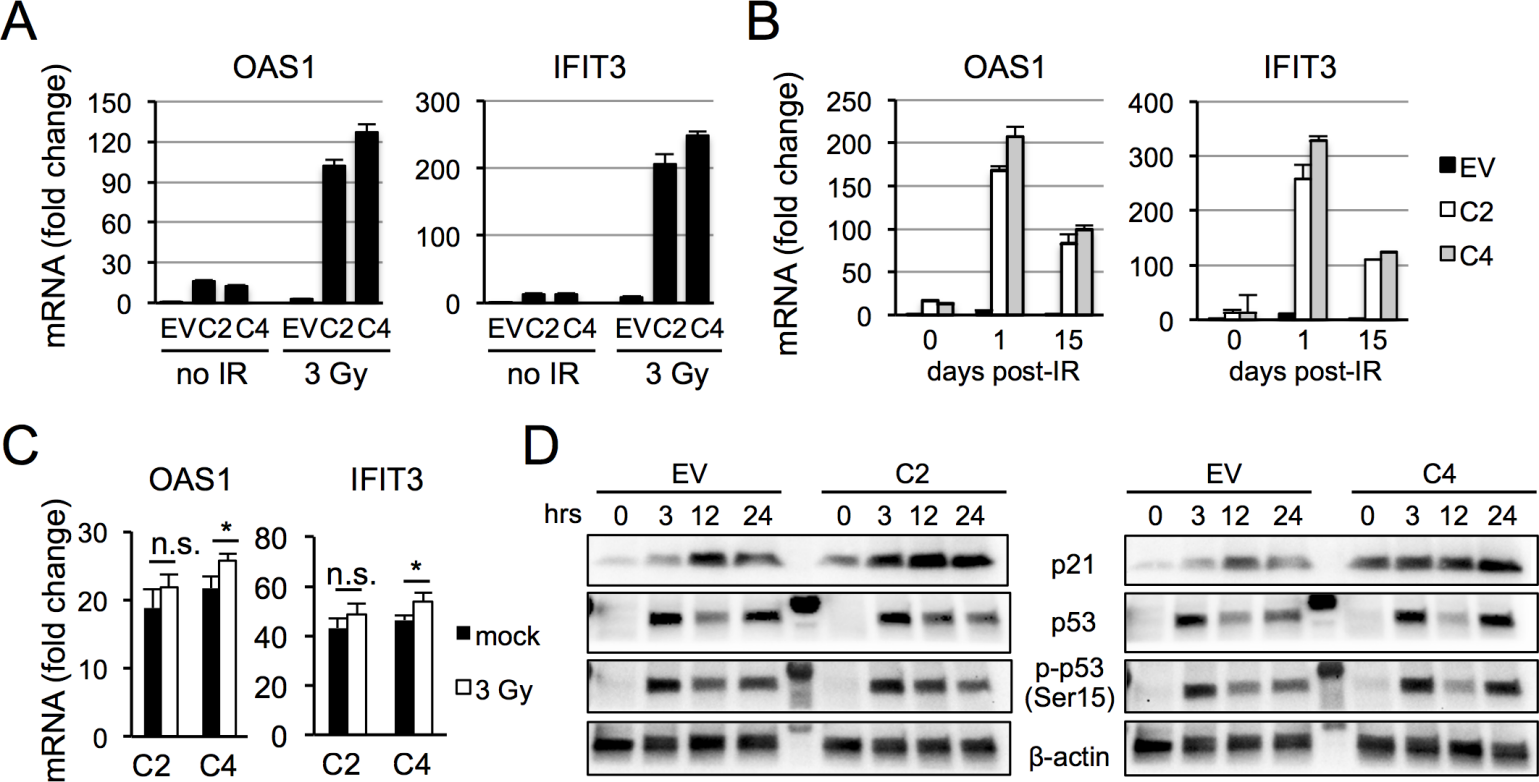
PARP1 depletion and radiation synergistically induce ISGs and p21 expression. (A) HCT116^EV^ and HCT-116^PARP1-/-^ cells (clones C2 and C4) treated with IR (3 Gy) or control nonirradiated cells were harvested and mRNA for ISGs OAS1 and IFIT3 quantified by qRT-PCR. Bars represent the average and standard deviation of quadruplicates. Data is representative of two independent experiments. (B) Cells treated as in (A) and unirradiated controls were passaged for 15 days and OAS1 and IFIT3 mRNA were quantified by qRT-PCR in unirradiated cells (t = 0) and in irradiated cells at 1 day and 15 days after IR. Data was normalized to the expression of unirradiated HCT116^EV^ cells (= 1). (C) Cells were incubated with the JAK inhibitor ruxolitinib and OAS1 and IFIT3 mRNAs were quantified by qRT-PCR after radiation and in control mock-irradiated cells. (D) Extracts from cells treated as in (A) were probed with antibodies to p21, p53 or phospho-p53 (Ser15) at the indicated timepoints (hours after radiation). As a loading control, the same blots were probed with an antibody to β-actin. Lane 5, molecular weight marker.

To determine whether IFNs contribute to the radiosensitivity of HCT116^*PARP1*-/-^ cells, we assessed the effect of PARP1 loss and radiation on the expression of ISGs OAS1 and IFIT3 (Fig 6). As expected, either PARP1 depletion or radiation was sufficient to induce ISG expression (Fig 6A). Strikingly, combined PARP1 depletion and radiation led to a synergistic increase in ISG expression (Fig 6A). ISG induction in PARP1-deficient cells was prolonged, with persistent levels even 15 days after a single dose of IR (Fig 6B) and was abrogated by pre-treatment of cells with the JAK inhibitor ruxolitinib (Fig 6C), suggesting a requirement for the IFN receptor. Mechanistically, PARP1 depletion and radiations also synergized in p21 induction (Fig 6D). In contrast, although p53 was stabilized and phosphorylated at Ser15 by radiation, we observed no significant differences between PARP1-proficient and deficient cells (Fig 6D).

## Discussion

It has long been known that nonviral nucleic acids can induce type I interferons [37]. However, the molecular basis of this phenomenon and its significance to human disease is only starting to be understood. Here, we show for the first time that PARP1, a ubiquitous factor with pleiotropic functions in DNA repair, transcription and other cellular processes, normally suppresses viral mimicry in human cells. Cells surviving PARP1 depletion evolve a stable, long-term pro-survival transcriptional program that requires continued RNA sensing/signaling via RIG-I/MAVS and amplification via an autocrine/paracrine loop involving the type I interferon receptor. This program functions to control cell proliferation, senescence and radiation responses.

Mice deficient for PARP1 are viable and, in the absence of stress, show only modest defects in growth [23]. Accordingly, our findings here and those of others [38, 39] clearly indicate that PARP1 is dispensable for survival of human cells. However, its loss significantly compromises human cell function even in the absence of exogenous stress. In this context, the most striking characteristic of cells surviving PARP1 depletion is their rewiring of the factors that regulate cell cycle progression, resulting in marked induction of p21 regardless of p53 status (active in HCT116 cells and inactive in HEK293T cells). This stable program is maintained via low level, constitutive type I interferon receptor signaling, a well-known inducer of growth arrest [40]. Our findings are in line with previous reports that cells surviving exposure to DNA damaging agents similarly activate an interferon-dependent transcriptional program that confers an anti-apoptotic function [41]. These phenotypes observed in long-term survivors are in contrast to the well-established roles for type I interferons in inducing cytotoxicity during acute exposure to DNA damaging agents [42, 43]. Therefore, understanding the mechanisms underlying the rewiring of interferon receptor signaling from a pro-death to a pro-survival program will be important to understand clonal evolution and to optimize the clinical use of drugs targeting innate immunity.

Our work also provides novel insights into the triggers of innate immune signaling, a particularly obscure area in the field [5]. In some settings, such as ATM deficiency [11] or exposure to IR [6], innate immunity is triggered by cGAS-dependent recognition of broken DNA, leading to the production of cGAMP and stimulation of STING. However, RNA sensing pathways also play roles in the activation of type I IFN after IR. Among RNA sensors, RIG-I senses dsRNAs with 5’-triphosphorylated (5’-PPP) or 5’-diphosphorylated (5’-PP) structures [44, 45] and, once activated, forms a complex with MAVS at the outer mitochondrial membrane to trigger signaling cascades that induce IFNs [46]. Loss of RIG-I or MAVS impairs the radiation-induced type I IFN response [7]. Here, we find that both RIG-I and MAVS are similarly required to maintain sustained ISG signaling in cells surviving PARP1 depletion. Although we do not yet know the dsRNA species that trigger RIG-I signaling in our system, several previous reports have implicated noncoding RNAs in RIG-I activation. Retroelements represent a potential candidate in PARP1-deficient cells because they are activated in response to DNA damage [47, 48] and promote innate immune signaling (reviewed in [49–51]). Moreover, endogenous retrovirus (ERVs) specifically activate RIG-I-dependent signaling in B cells [10]. Other candidates include U1 and U2 RNAs, which stimulate innate immune signaling after IR [7] or UV [52]. Interestingly, a recent report uncovered that, although many noncoding RNAs are present in the cytoplasm of breast cancer cells, a single RNA, RN7SL1, was sufficient to activate RIG-I signaling [14]. In the future, it will be interesting to determine whether lineage-specific noncoding RNAs may similarly underlie RIG-I activation in response to PARP1 depletion.

Consistent with our findings that RIG-I is functionally significant, we observed a dramatic induction of the RIG-I protein in PARP1-deficient cells. In contrast, RIG-I mRNA was induced only about two-fold, suggesting that this factor is regulated mostly via post-translational modification [53]. Significantly, the mRNA and, to a much greater extent, the protein level for STING, MDA-5, and TLR3 were also markedly increased in PARP1-deficient cells, even though efficient knock down of these factors had no effect on ISG expression. These findings may result from the incomplete nature of the knock down, but could also reflect on activation of these factors via cross-talk with RIG-I or other pathways [54] or, in the case of STING, defective translocation in the absence of cGAS expression [34]. In the future, it will be interesting to determine whether cGAS-independent pathways [55, 56] may activate STING upon PARP1 loss and how cytoplasmic sensors interacts with each other to regulate specific IFN targets and cellular outcomes specifically in PARP1-depleted cells [57, 58].

In summary, we have generated and validated two cell line-based models to investigate PARP1 functions in SSB repair, radiation responses and innate immune signaling. Given PARP1 pleiotropic roles in aging, inflammation, degeneration and cancer [59–61], these systems may provide useful reagents for the future development of PARP inhibitors and other drugs that target innate immune signaling for the prevention and/or treatment of these disorders. Finally, we find that loss of PARP1 modulates the expression of PDL1 and other molecules involved in adaptive immunity (S1 Table), and future development of models that examine the crosstalk between the innate and adaptive immune systems [62, 63] upon PARP1 loss or inhibition is warranted by these data.

## Supporting information

S1 Fig Strategy for PARP1 locus inactivation via CRISPR/Cas9^D10A^ “double nicking”. (A) Sequence of gRNAs A and B binding to sequences within exon 2 of PARP1, downstream of the ATG. (B) To quantify the efficiency of genome editing, both gRNAs A and B were expressed together with Cas9^D10A^ (“double nicking”) or individually with Cas9 (“nuclease”) in HEK293T cells. After puromycin selection, the target region was amplified from genomic DNA and editing was measured via the T7 assay. Black arrow points to the unedited (wild-type) band. Lower molecular bands of the expected size were observed. (C) Editing of the target region was confirmed by Sanger sequencing of the target region after TOPO-TA cloning of PCR products. The consensus sequence is shown on top. (D) Expression of either gRNA A or B with Cas9^D10A^ did not result in genome modification in the T7 assay.
(PDF)

S2 Fig Generation of HCT116 cells deficient for PARP1 (HCT116^*PARP1-/-*^) via”double nicking” at PARP1 exon 2. (A) gRNAs A and B were expressed together with Cas9^D10A^ in HCT-116 cells. After puromycin selection, the efficiency of “double nicking” was measured via the T7 assay. Black arrow, unmodified band; red arrows, modified products. (B) After single cell subcloning and immunoblotting for PARP1, clones C2 and C4 were selected for further analysis. To ensure clonality, gDNA was amplified and PCR products were cloned into TOPO-TA and sequenced. The modified alleles are expected to yield a truncated mRNA, consistent with lack of protein expression. The consensus sequence is shown on top.
(PDF)

S3 Fig HEK293T cells deficient for PARP1 (HEK293^*PARP1*-/-^ cells) are viable but growth retarded. (A) After targeting of PARP1 exon 2 using the “double nickase” approach, single cell-derived subclones were expanded and extracts immunoblotted with an antibody to PARP1. Knock out (KO) clones are indicated by red asterisks. EV, extracts from cells transfected with an “empty vector” expressing Cas9^D10A^ but no gRNAs. (B) Cell proliferation for 6 KO clones and control EV cells. 10^5^ cells were seeded in 150 mm plates and counted at day 10. (C-D) Cloning efficiency of KO clones C5 and C9 relative to control EV cells. Bars in (C) represent the average and standard deviation of 3 plates, normalized to EV. Representative examples are shown in (D). (E-F) Xenograft assay of PARP1 KO clone C5 relative to control EV cells. Cells were injected in the flanks of NGS mice and tumor volume measured at the indicated timepoints. Graphs in (E) represent the average and standard deviation of 5 tumors per line. Representative tumors at day 20 are shown in (F). (G) Example of senescent cells (positive for SA-associated β-galactosidase; blue) in PARP1-deficient cultures (clone C5). (H-I) Cell cycle analysis of six KO clones and control EV cells. The percentage of cells with 4N DNA content in shown. Bars in (H) represent the average and standard deviation of 3 independent experiments. Representative examples are shown in (I). (J) Analysis of RNA-Seq data for members of the CDKN2 and CDKN1 families. (K) Extracts from PARP1-deficient cells (clones C5 and C9) and control EV cells were probed with antibodies to p21, p53 and, as a loading control, GAPDH.
(PDF)

S4 Fig PARP1 reconstitution partially rescues phenotypes of HEK293T^*PARP1*-/-^ cells. (A) After infection with a lentiviral vector containing the PARP1 cDNA or a control vector lacking cDNA insert, PARP1 expression was quantified by immunoblotting. The level in reconstituted cells was compared to the endogenous levels in HCT116^EV^ cells (lane 1). GAPDH, loading control. (B) To confirm that the reconstituted PARP1 protein was active, extracts from reconstituted cells were probed with an antibody to PAR in baseline conditions or 15 minutes after exposure to IR (to induce PARylation). GAPDH, loading control. (C-D) PARP1 reconstitution partially rescues resistance to the PARP inhibitor olaparib in PARP1-deficient cells. Cells counts after exposure to 5 μM olaparib or vehicle (DMSO) for 48 hours revealed that PARP1-deficient clones C5 and C9 are olaparib-resistant (C). Upon PARP1 re-expression, sensitivity to olaparib was partially restored (D). Bars represent the average and standard deviation of triplicates. Data is representative of two independent experiments. (E-F) PARP1 reconstitution did not significantly rescue defects in proliferation in PARP1-deficient cells. A proliferation assay of PARP1-deficient and reconstituted cells is shown in (E). Bars represent the average and standard deviation of triplicates. The experiment is representative of two independent experiments. Cell cycle analysis of PARP1-deficient C9 cells and PARP1-reconstituted C9 cells is shown in (F). The percentage of cells with 4N DNA content is indicated.
(PDF)

S5 Fig Transient PARP1 reconstitution partially rescues growth and cell cycle defects in HCT116^*PARP1*-/-^ cells. (A-B) HCT116 cells were transfected with a plasmid expressing GFP-PARP1. A representative image of the culture 48 hours after transfection is shown in (A). To quantify PARP1 expression in reconstituted cells, extracts of PARP1-reconstituted and parental PARP1 null cells (clones C2 and C4) were hybridized with an antibody to PARP1 (B). The endogenous and the higher molecular weight GFP-PARP1 species are indicated. For comparison, the endogenous level of PARP1 is shown in lane 1 (HCT116^EV^ cells). GAPDH, loading control. (C-D) PARP1 reconstitution partially rescues resistance to the PARPi olaparib in PARP1-deficient cells. Control HCT116^EV^ or PARP1-deficient clones C2 and C4 were treated with 5 μM olaparib or vehicle (DMSO) for 48 hours and cells were counted (C). To assess the effect of reconstitution on olaparib sensitivity, reconstituted cells were treated with 5 μM olaparib 3 days after transfection and counted after 48 hours of continuous treatment (D). For all graphs, bars represent the average and standard deviation of triplicates or quadruplicates. Data is representative of two independent experiments. (E) Cell counts of null and PARP1-reconstituted HCT116^*PARP1*-/-^ cells, clones C2 and C4, at day 7 after transfection.
(PDF)

S6 Fig Quantification of chromosomal breaks in HCT116 cells surviving PARP1 depletion. Exponentially growing HCT116^*PARP1*-/-^ cells (clones C2 and C4) and control HCT116^EV^ cells were incubated in colcemid, fixed and metaphase spreads were hybridized with a PNA probe that binds to telomere sequences (TTAGGG; red). DNA was counterstained with DAPI (blue). Representative metaphases are shown.
(PDF)

S7 Fig PARP1-deficient cells are hypersensitive to MMS. (A) HCT116^*PARP1*-/-^ cells (clones C2 and C4) and control PARP1-proficient HCT116^EV^ cells were treated with 0.01% MMS for 2 hours and cells were counted after 48 hours. Bars represent the average and standard deviation of triplicate plates per experiment, pooled from 2 independent experiments. (B) HCT116^*PARP1*-/-^ cells (clones C2 and C4) and control PARP1-proficient HCT116^EV^ cells were treated with 0.05% MMS for 2 hours cells were counted after 48 hours. Bars represent the average and standard deviation of triplicate plates. Data is representative of 3 independent experiments.
(PDF)

S8 Fig The repair of IR-dependent DSBs is delayed in HEK293T^PARP1-/-^ cells. (A) Cell cycle distribution of HEK293T^PARP1-/-^ cells (clones C5 and C9) and control, PARP1-proficient HEK293T^EV^ cells at the indicated timepoints after exposure to IR (5 Gy). The percentage of cells with 2N and 4N DNA content is indicated. (B) HEK293T^PARP1-/-^ cells (clones C5 and C9) and control HEK293T^EV^ cells were exposed to IR (5 Gy) and extracts were harvested at the indicated timepoints after IR and probed with antibodies to gamma-H2AX, phospho-KAP1 (Ser824) and phospho-CHK2 (Thr68).
(PDF)

S9 Fig Analysis of RNA-Seq data. (A) Volcano plots of fold change (x axis) *vs*. p value (y axis). Data from 2 independent PARP1 null clones per line was compared to the empty vector (EV) control line. Red, induced mRNAs; blue, repressed mRNAs. Blue arrow points to PARP1 mRNA. (B) Scatter plot analysis of mRNAs differentially expressed in HCT116 ^*PARP1*-/-^ cells vs. HEK293T^*PARP1*-/-^ cells.
(PDF)

S10 Fig Gene Ontology (GO) analyses of RNA-Seq data. (A) mRNAs induced in HCT116^*PARP1*-/-^ cells (clones C2 and C4) relative to HCT116^EV^ cells were prominently enriched for Molecular Function: “binding” (level 1); “protein binding” (level 2” and “receptor binding” (level 3). (B) Enrichment for the same hierarchy was observed in HEK293T^*PARP1*-/-^ cells (clones C5 and C9) relative to HEK293T^EV^ cells.
(PDF)

S11 Fig Analysis of type I IFN pathway in HEK293T^*PARP1*-/-^ cells relative to control HEK293T^EV^ cells. (A-B) RNA-Seq data was analyzed using GSEA. The enrichment plot for “Interferon Alpha Response” genes is shown in (A). A heatmap showing differential expression for individual genes in this category is shown in (B). (C) Quantification of mRNA for 12 interferon-inducible genes (ISGs) in HEK293T^EV^ HEK293T^*PARP1*-/-^ (clones C5 and C9).
(PDF)

S12 Fig Comparison of mRNA expression for 32 ISGs in HEK293T^EV^ and HCT116^EV^ cells. ISG mRNA was quantified by RT-PCR in parallel for the two lines and normalized to ribosomal RNA18S. The expression of each mRNA in HEK293T^EV^ cells was normalized to the expression of the same mRNA for HCT116 ^EV^ cells. Values below 1 indicate higher expression in HCT116^EV^ cells; values higher than 1 indicate higher expression in HEK293T^EV^ cells.
(PDF)

S13 Fig Expression of factors involved in the detection and signaling of cytoplasmic nucleic acids in HEK293T cells surviving PARP1 depletion. Extracts from control HEK293T^EV^ cells and HEK293^*PARP1*-/-^ cells (clones C5 and C9) were hybridized with antibodies to MDA5 (A), RIG-I (A), MAVS (A), TLR3 (B) and STING (B). Extracts from HCT116^EV^ and HCT116^PARP1-/-^ cells (clones C2 and C4) were analyzed in parallel for direct comparison. GAPDH, loading control.
(PDF)

S14 Fig Efficiency of “knock down” (KD) for the indicated factors involved in the sensing/signaling of cytoplasmic nucleic acids during viral mimicry. Cells were transfected with siRNA pools for each factor (100 nM) and mRNA expression was quantified by q-RT-PCR 4 days after transfection. All values are normalized to the expression in untreated HCT116^EV^ cells (= 1). Bars represent the average and standard deviation of quadruplicates. Data is representative of two independent experiments.
(PDF)

S1 Table RNA-Seq analysis of HCT116^*PARP1*-/-^ cells. (XLSX)

S2 Table RNA-Seq analysis of HEK293T^*PARP1*-/-^ cells. (XLSX)

S3 Table Gene Set Enrichment Analysis (GSEA; Hallmark) of pathways differentially expressed in HCT116^PARP1-/-^ and HCT-116^EV^ cells. (PDF)

S4 Table Gene Set Enrichment Analysis (GSEA; Hallmark) of pathways differentially expressed in HEK293T^PARP1-/-^ and HEK293T^EV^ cells. (PDF)

S5 Table Canonical pathways differentially regulated in HCT116^*PARP1*-/-^ cells relative to HCT116^EV^ cells (IPA). (PDF)

S6 Table Canonical pathways differentially regulated in HEK293T^PARP1-/-^ cells relative to HEK293^EV^ cells (IPA). (PDF)

S7 Table List of primers used in the study and literature references if applicable. (PDF)
