# Supplementary figures and images for: PARP1 depletion induces RIG-I-dependent signaling in human cancer cells
Source: PLoS One. 2018 Mar 28;13(3):e0194611. doi: 10.1371/journal.pone.0194611 (PMC5874037; doi:10.1371/journal.pone.0194611)

A

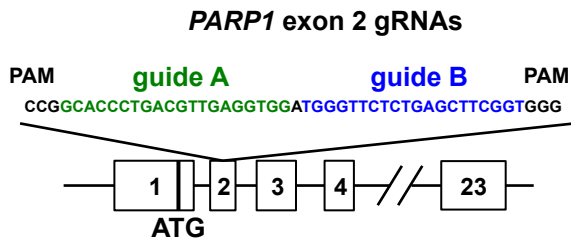

B

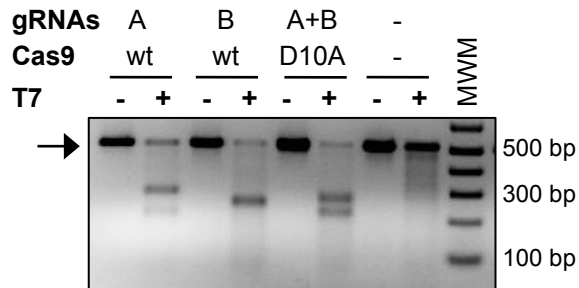

C

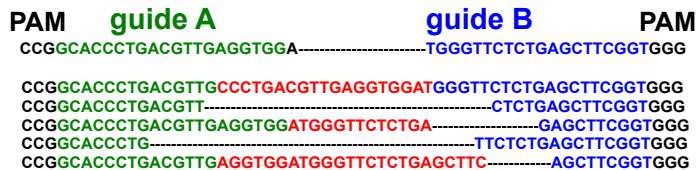

D

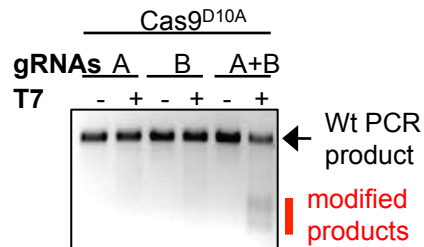

Supplement: S1 Fig — (A) Sequence of gRNAs A and B binding to sequences within exon 2 of PARP1, downstream of the ATG. (B) To quantify the efficiency of genome editing, both gRNAs A and B were expressed together with Cas9D10A (“double nicking”) or individually with Cas9 (“nuclease”) in HEK293T cells. After puromycin selection, the target region was amplified from genomic DNA and editing was measured via the T7 assay. Black arrow points to the unedited (wild-type) band. Lower molecular bands of the expected size were observed. (C) Editing of the target region was confirmed by Sanger sequencing of the target region after TOPO-TA cloning of PCR products. The consensus sequence is shown on top. (D) Expression of either gRNA A or B with Cas9D10A did not result in genome modification in the T7 assay. (PDF) [file pone.0194611.s001.pdf]

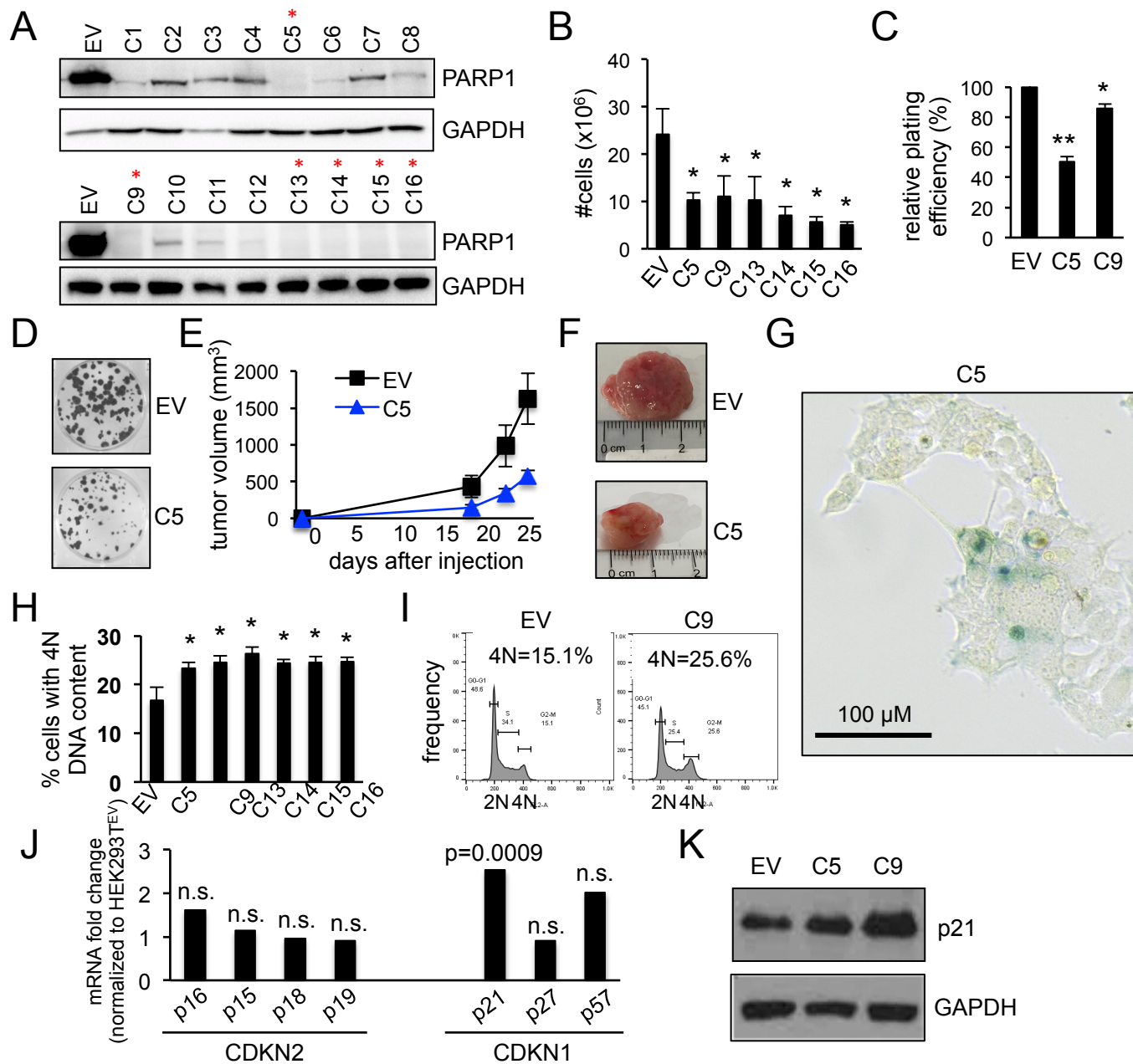

Supplement: S3 Fig — (A) After targeting of PARP1 exon 2 using the “double nickase” approach, single cell-derived subclones were expanded and extracts immunoblotted with an antibody to PARP1. Knock out (KO) clones are indicated by red asterisks. EV, extracts from cells transfected with an “empty vector” expressing Cas9D10A but no gRNAs. (B) Cell proliferation for 6 KO clones and control EV cells. 105 cells were seeded in 150 mm plates and counted at day 10. (C-D) Cloning efficiency of KO clones C5 and C9 relative to control EV cells. Bars in (C) represent the average and standard deviation of 3 plates, normalized to EV. Representative examples are shown in (D). (E-F) Xenograft assay of PARP1 KO clone C5 relative to control EV cells. Cells were injected in the flanks of NGS mice and tumor volume measured at the indicated timepoints. Graphs in (E) represent the average and standard deviation of 5 tumors per line. Representative tumors at day 20 are shown in (F). (G) Example of senescent cells (positive for SA-associated β-galactosidase; blue) in PARP1-deficient cultures (clone C5). (H-I) Cell cycle analysis of six KO clones and control EV cells. The percentage of cells with 4N DNA content in shown. Bars in (H) represent the average and standard deviation of 3 independent experiments. Representative examples are shown in (I). (J) Analysis of RNA-Seq data for members of the CDKN2 and CDKN1 families. (K) Extracts from PARP1-deficient cells (clones C5 and C9) and control EV cells were probed with antibodies to p21, p53 and, as a loading control, GAPDH. (PDF) [file pone.0194611.s003.pdf]

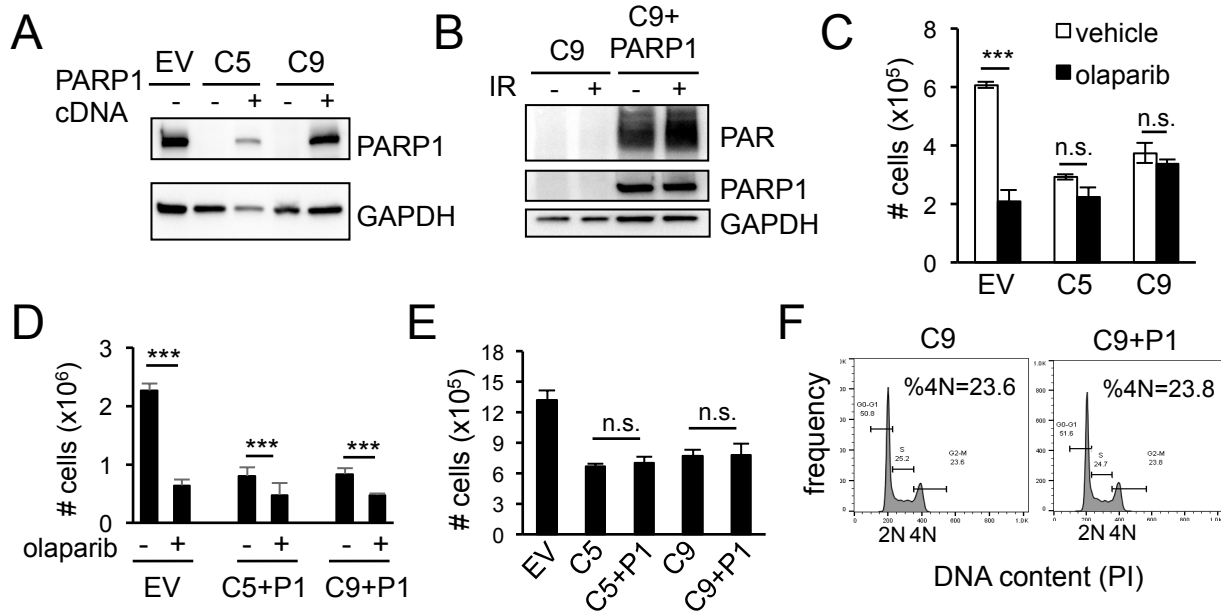

Supplement: S4 Fig — (A) After infection with a lentiviral vector containing the PARP1 cDNA or a control vector lacking cDNA insert, PARP1 expression was quantified by immunoblotting. The level in reconstituted cells was compared to the endogenous levels in HCT116EV cells (lane 1). GAPDH, loading control. (B) To confirm that the reconstituted PARP1 protein was active, extracts from reconstituted cells were probed with an antibody to PAR in baseline conditions or 15 minutes after exposure to IR (to induce PARylation). GAPDH, loading control. (C-D) PARP1 reconstitution partially rescues resistance to the PARP inhibitor olaparib in PARP1-deficient cells. Cells counts after exposure to 5 μM olaparib or vehicle (DMSO) for 48 hours revealed that PARP1-deficient clones C5 and C9 are olaparib-resistant (C). Upon PARP1 re-expression, sensitivity to olaparib was partially restored (D). Bars represent the average and standard deviation of triplicates. Data is representative of two independent experiments. (E-F) PARP1 reconstitution did not significantly rescue defects in proliferation in PARP1-deficient cells. A proliferation assay of PARP1-deficient and reconstituted cells is shown in (E). Bars represent the average and standard deviation of triplicates. The experiment is representative of two independent experiments. Cell cycle analysis of PARP1-deficient C9 cells and PARP1-reconstituted C9 cells is shown in (F). The percentage of cells with 4N DNA content is indicated. (PDF) [file pone.0194611.s004.pdf]

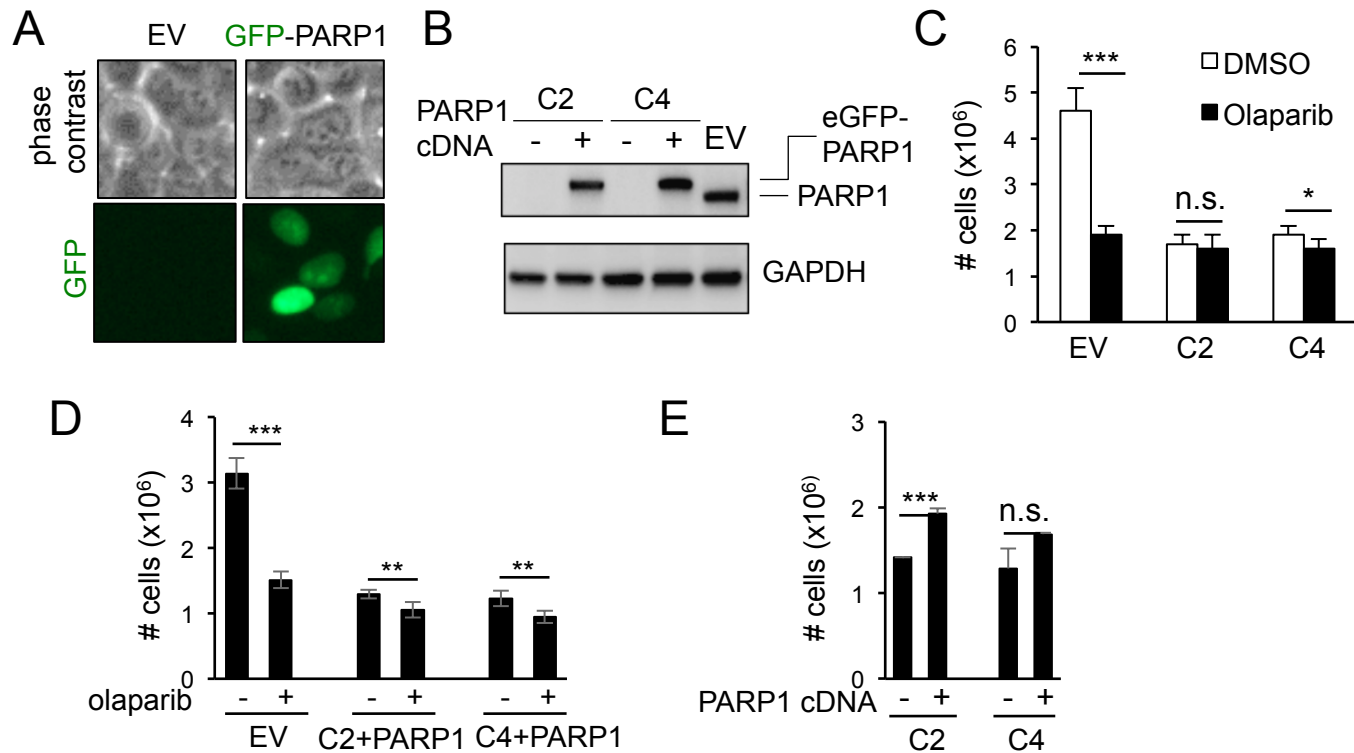

Supplement: S5 Fig — (A-B) HCT116 cells were transfected with a plasmid expressing GFP-PARP1. A representative image of the culture 48 hours after transfection is shown in (A). To quantify PARP1 expression in reconstituted cells, extracts of PARP1-reconstituted and parental PARP1 null cells (clones C2 and C4) were hybridized with an antibody to PARP1 (B). The endogenous and the higher molecular weight GFP-PARP1 species are indicated. For comparison, the endogenous level of PARP1 is shown in lane 1 (HCT116EV cells). GAPDH, loading control. (C-D) PARP1 reconstitution partially rescues resistance to the PARPi olaparib in PARP1-deficient cells. Control HCT116EV or PARP1-deficient clones C2 and C4 were treated with 5 μM olaparib or vehicle (DMSO) for 48 hours and cells were counted (C). To assess the effect of reconstitution on olaparib sensitivity, reconstituted cells were treated with 5 μM olaparib 3 days after transfection and counted after 48 hours of continuous treatment (D). For all graphs, bars represent the average and standard deviation of triplicates or quadruplicates. Data is representative of two independent experiments. (E) Cell counts of null and PARP1-reconstituted HCT116PARP1-/- cells, clones C2 and C4, at day 7 after transfection. (PDF) [file pone.0194611.s005.pdf]

HCT116<sup>EV</sup>

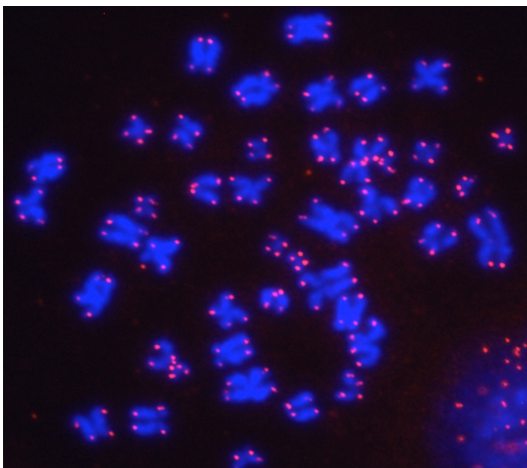

HCT116<sup>PARP1-/-</sup>  
clone C2

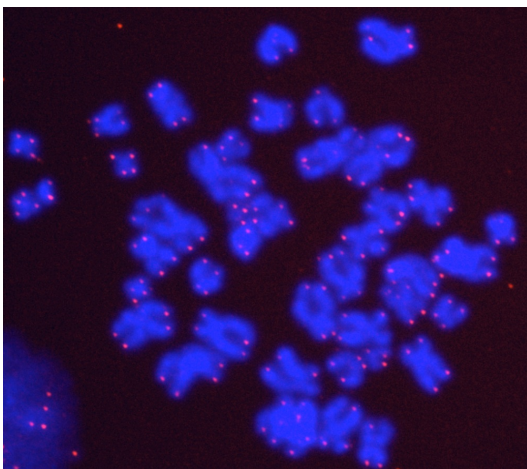

HCT116<sup>PARP1-/-</sup>  
clone C4

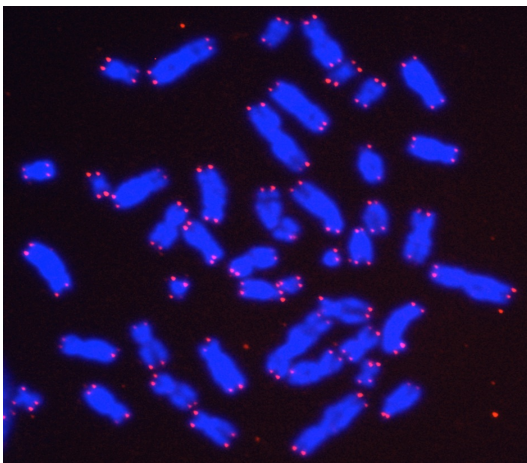

DAPI (TTAGGG)<sub>3</sub>

Supplement: S6 Fig — Exponentially growing HCT116PARP1-/- cells (clones C2 and C4) and control HCT116EV cells were incubated in colcemid, fixed and metaphase spreads were hybridized with a PNA probe that binds to telomere sequences (TTAGGG; red). DNA was counterstained with DAPI (blue). Representative metaphases are shown. (PDF) [file pone.0194611.s006.pdf]

**A****HCT116**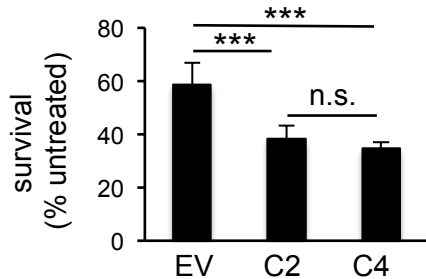**B****HEK293T**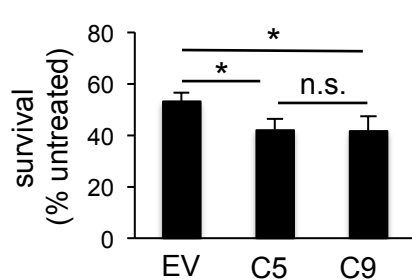

Supplement: S7 Fig — (A) HCT116PARP1-/- cells (clones C2 and C4) and control PARP1-proficient HCT116EV cells were treated with 0.01% MMS for 2 hours and cells were counted after 48 hours. Bars represent the average and standard deviation of triplicate plates per experiment, pooled from 2 independent experiments. (B) HCT116PARP1-/- cells (clones C2 and C4) and control PARP1-proficient HCT116EV cells were treated with 0.05% MMS for 2 hours cells were counted after 48 hours. Bars represent the average and standard deviation of triplicate plates. Data is representative of 3 independent experiments. (PDF) [file pone.0194611.s007.pdf]

A

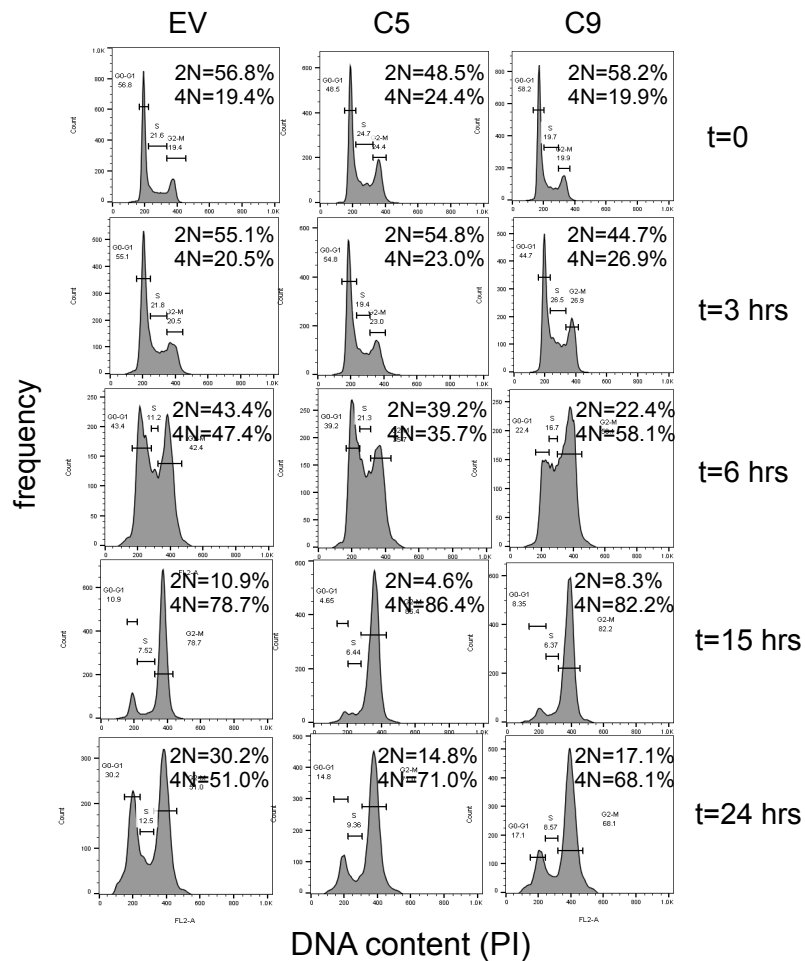

B

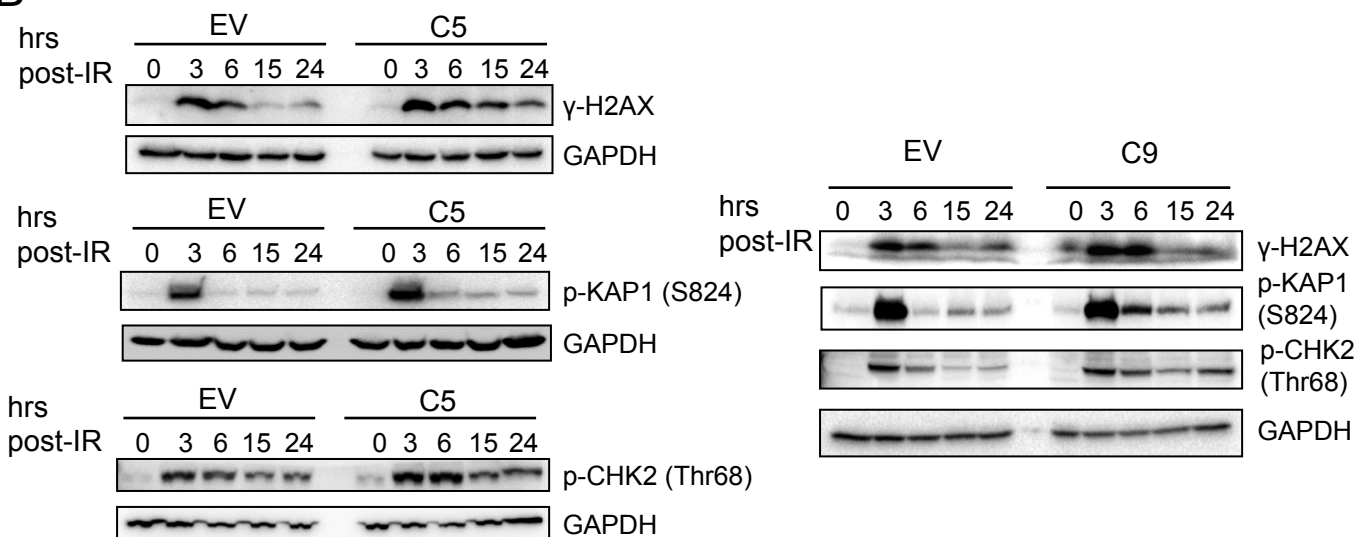

Supplement: S8 Fig — (A) Cell cycle distribution of HEK293TPARP1-/- cells (clones C5 and C9) and control, PARP1-proficient HEK293TEV cells at the indicated timepoints after exposure to IR (5 Gy). The percentage of cells with 2N and 4N DNA content is indicated. (B) HEK293TPARP1-/- cells (clones C5 and C9) and control HEK293TEV cells were exposed to IR (5 Gy) and extracts were harvested at the indicated timepoints after IR and probed with antibodies to gamma-H2AX, phospho-KAP1 (Ser824) and phospho-CHK2 (Thr68). (PDF) [file pone.0194611.s008.pdf]

A

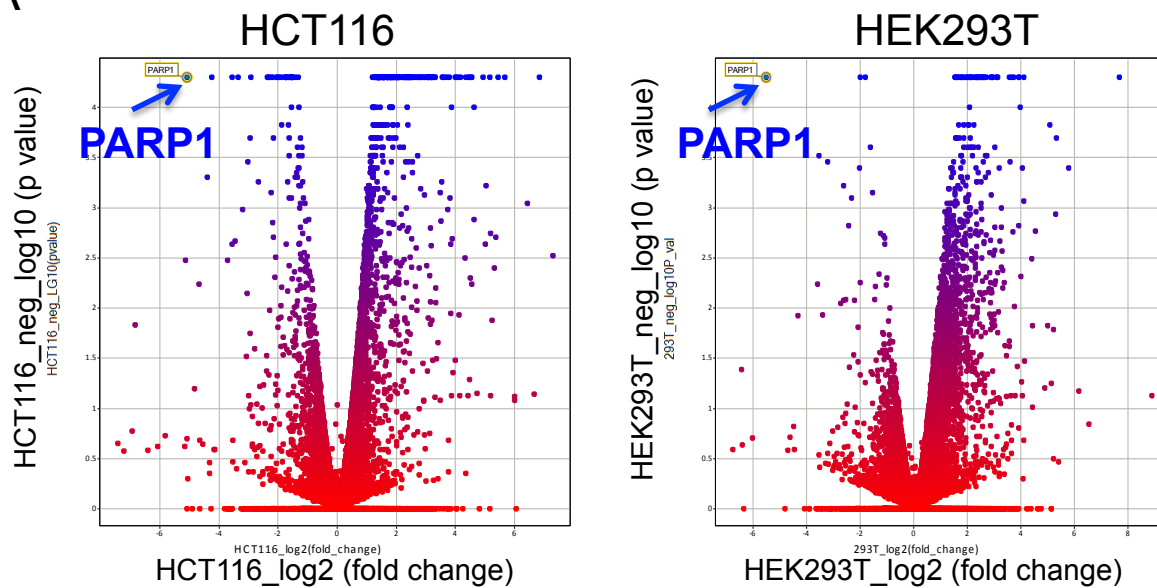

B

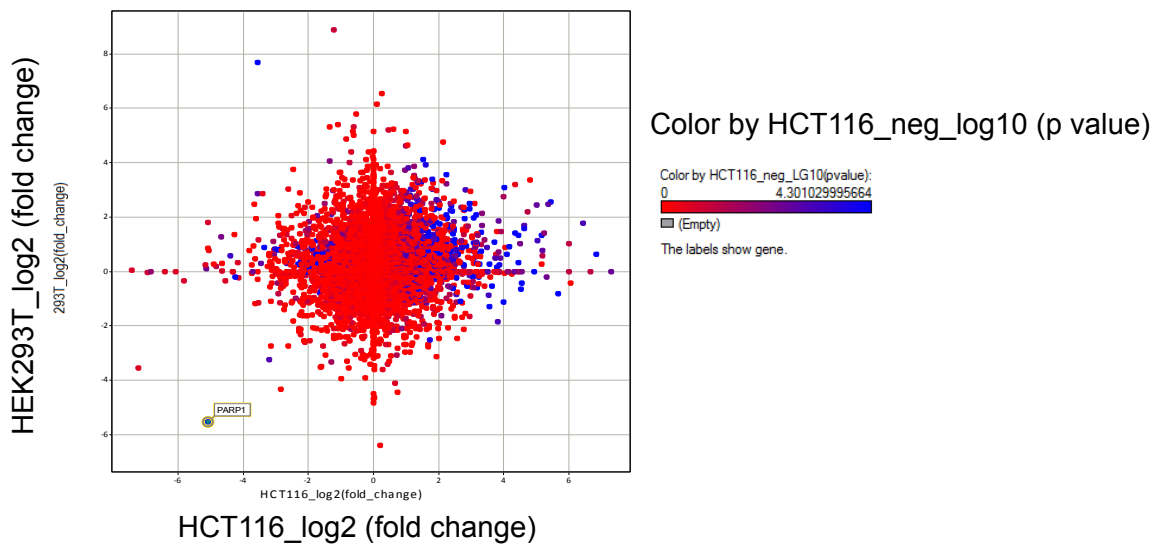

Supplement: S9 Fig — (A) Volcano plots of fold change (x axis) vs. p value (y axis). Data from 2 independent PARP1 null clones per line was compared to the empty vector (EV) control line. Red, induced mRNAs; blue, repressed mRNAs. Blue arrow points to PARP1 mRNA. (B) Scatter plot analysis of mRNAs differentially expressed in HCT116 PARP1-/- cells vs. HEK293TPARP1-/- cells. (PDF) [file pone.0194611.s009.pdf]

# B

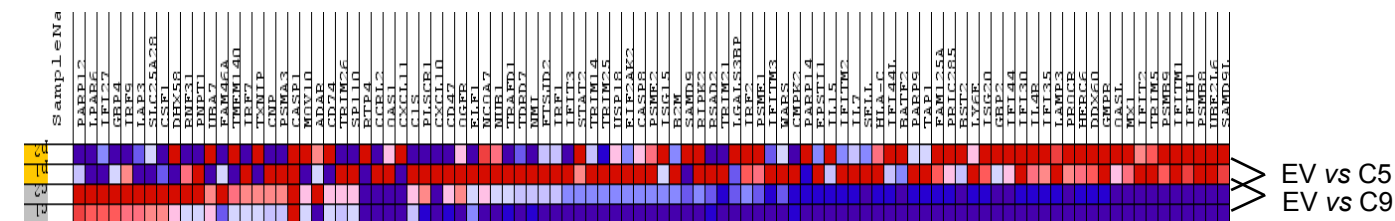

mRNA(fold change)

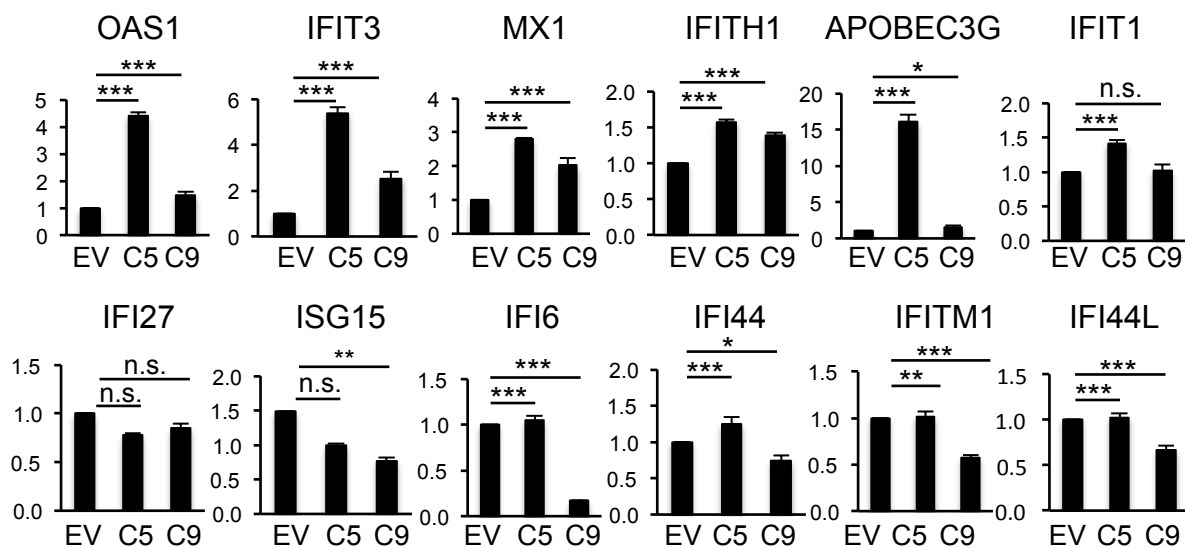

Supplement: S11 Fig — (A-B) RNA-Seq data was analyzed using GSEA. The enrichment plot for “Interferon Alpha Response” genes is shown in (A). A heatmap showing differential expression for individual genes in this category is shown in (B). (C) Quantification of mRNA for 12 interferon-inducible genes (ISGs) in HEK293TEV HEK293TPARP1-/- (clones C5 and C9). (PDF) [file pone.0194611.s011.pdf]

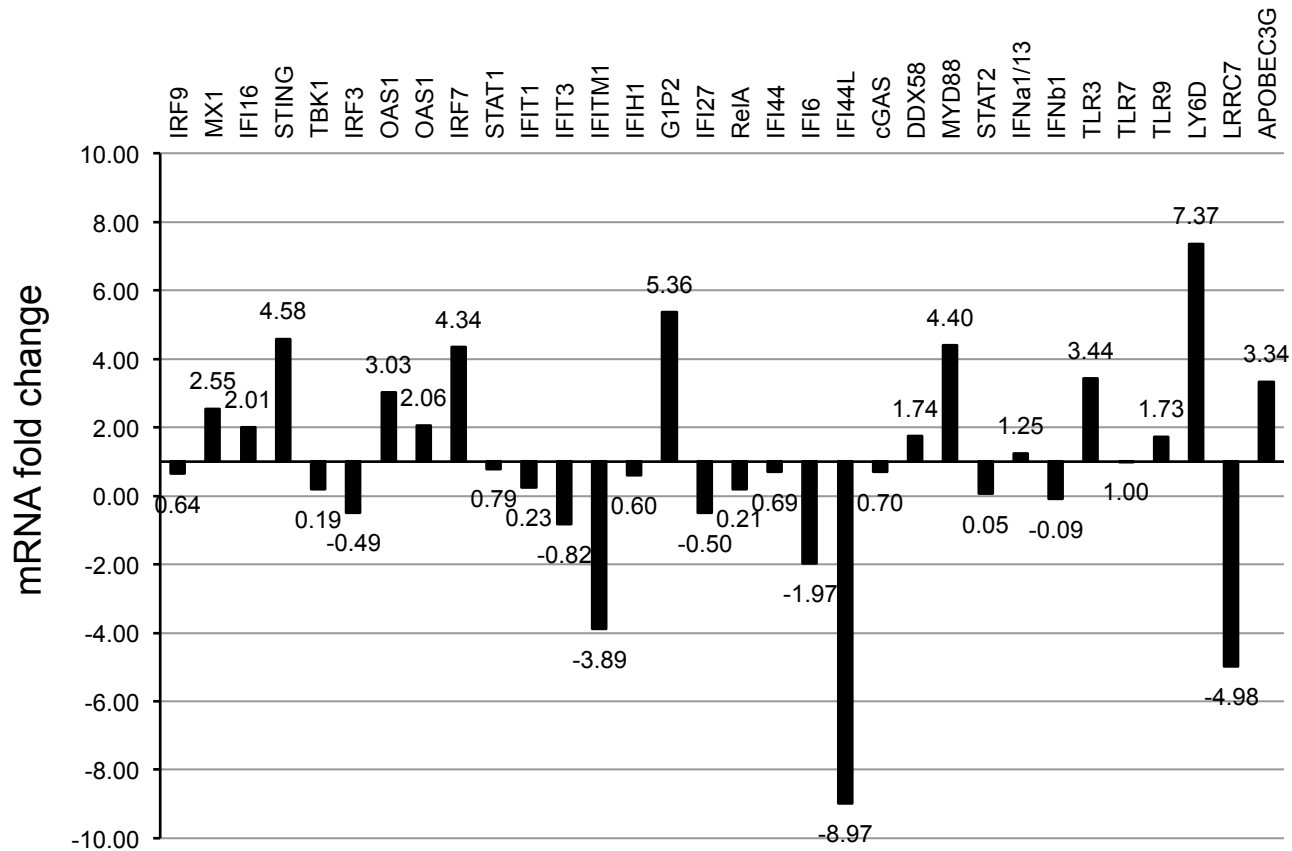

Supplement: S12 Fig — ISG mRNA was quantified by RT-PCR in parallel for the two lines and normalized to ribosomal RNA18S. The expression of each mRNA in HEK293TEV cells was normalized to the expression of the same mRNA for HCT116 EV cells. Values below 1 indicate higher expression in HCT116EV cells; values higher than 1 indicate higher expression in HEK293TEV cells. (PDF) [file pone.0194611.s012.pdf]

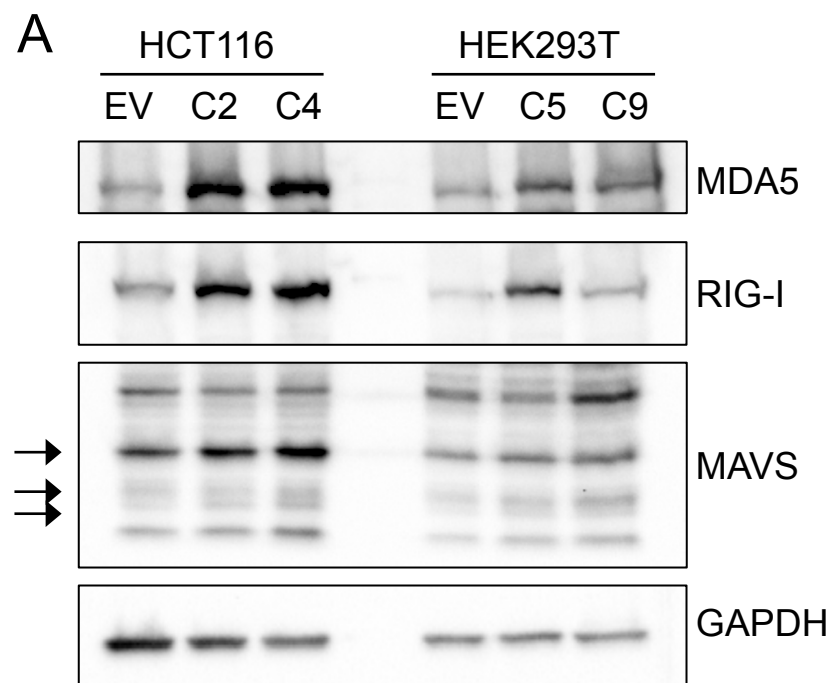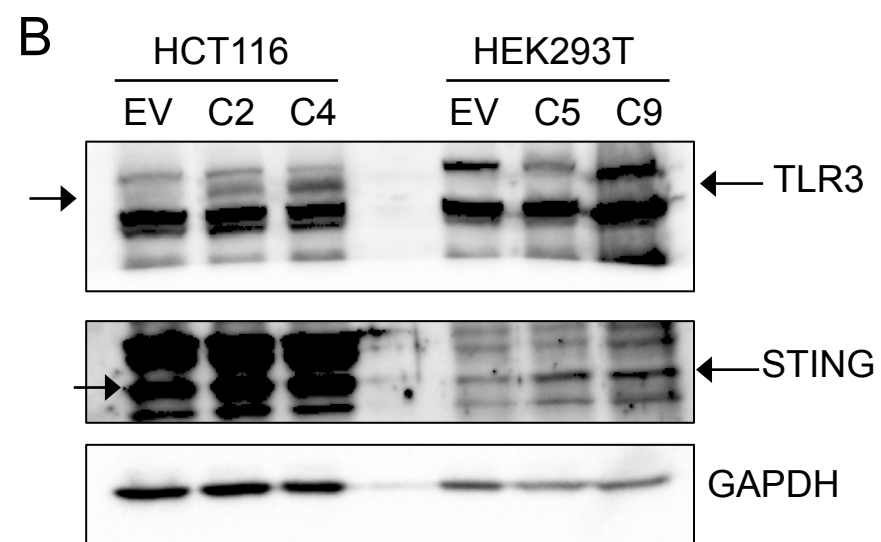

Supplement: S13 Fig — Extracts from control HEK293TEV cells and HEK293PARP1-/- cells (clones C5 and C9) were hybridized with antibodies to MDA5 (A), RIG-I (A), MAVS (A), TLR3 (B) and STING (B). Extracts from HCT116EV and HCT116PARP1-/- cells (clones C2 and C4) were analyzed in parallel for direct comparison. GAPDH, loading control. (PDF) [file pone.0194611.s013.pdf]

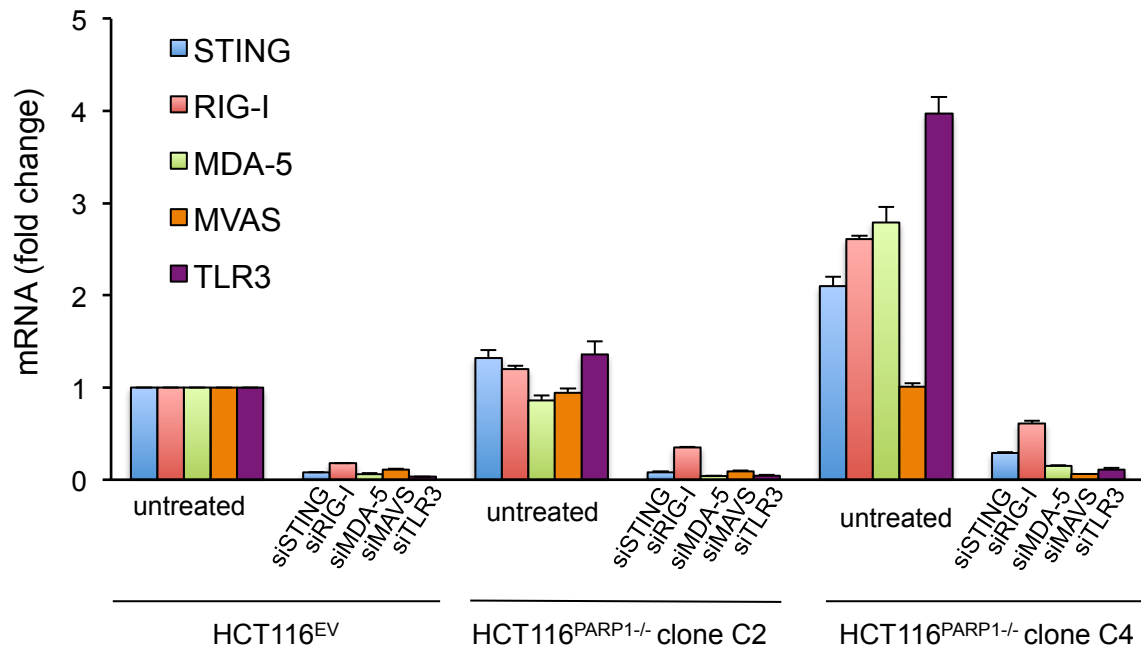

Supplement: S14 Fig — Cells were transfected with siRNA pools for each factor (100 nM) and mRNA expression was quantified by q-RT-PCR 4 days after transfection. All values are normalized to the expression in untreated HCT116EV cells (= 1). Bars represent the average and standard deviation of quadruplicates. Data is representative of two independent experiments. (PDF) [file pone.0194611.s014.pdf]
