# Supplementary material for: PARP1 depletion induces RIG-I-dependent signaling in human cancer cells
Source: PLoS One. 2018 Mar 28;13(3):e0194611. doi: 10.1371/journal.pone.0194611 (PMC5874037; doi:10.1371/journal.pone.0194611)
Supplement: S2 Fig — (A) gRNAs A and B were expressed together with Cas9D10A in HCT-116 cells. After puromycin selection, the efficiency of “double nicking” was measured via the T7 assay. Black arrow, unmodified band; red arrows, modified products. (B) After single cell subcloning and immunoblotting for PARP1, clones C2 and C4 were selected for further analysis. To ensure clonality, gDNA was amplified and PCR products were cloned into TOPO-TA and sequenced. The modified alleles are expected to yield a truncated mRNA, consistent with lack of protein expression. The consensus sequence is shown on top. (PDF) [file pone.0194611.s002.pdf]

A

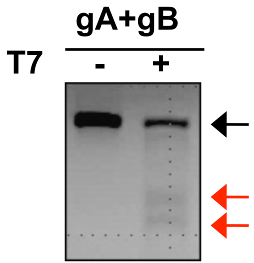

B

	PAM guide A guide B PAM
consensus	CCGGCACCCTGACGTTGAGGTGG A TGGGTTCTCTGAGCTTCGGTGGG
C2 allele 1	CCGGCACCCTGACGTTGAG--CTTCGGTGGG
C2 allele 2	CCGGCACCCTGACGTTGAGG-------------------GCTCTCTGAGCTTCACCCTGAGCTTCGGTGGG
C4 both alleles	CCGGCACCCTGACGT-------------------CAGCTGCGCGCTCGCTCGCTCACTGAGCTTCGGTGGG
