# Supplementary material for: PARP1 depletion induces RIG-I-dependent signaling in human cancer cells
Source: PLoS One. 2018 Mar 28;13(3):e0194611. doi: 10.1371/journal.pone.0194611 (PMC5874037; doi:10.1371/journal.pone.0194611)

A

HCT116

(induced in HCT116$^{PARP1-/-}$ relative to HCT116^{EV})

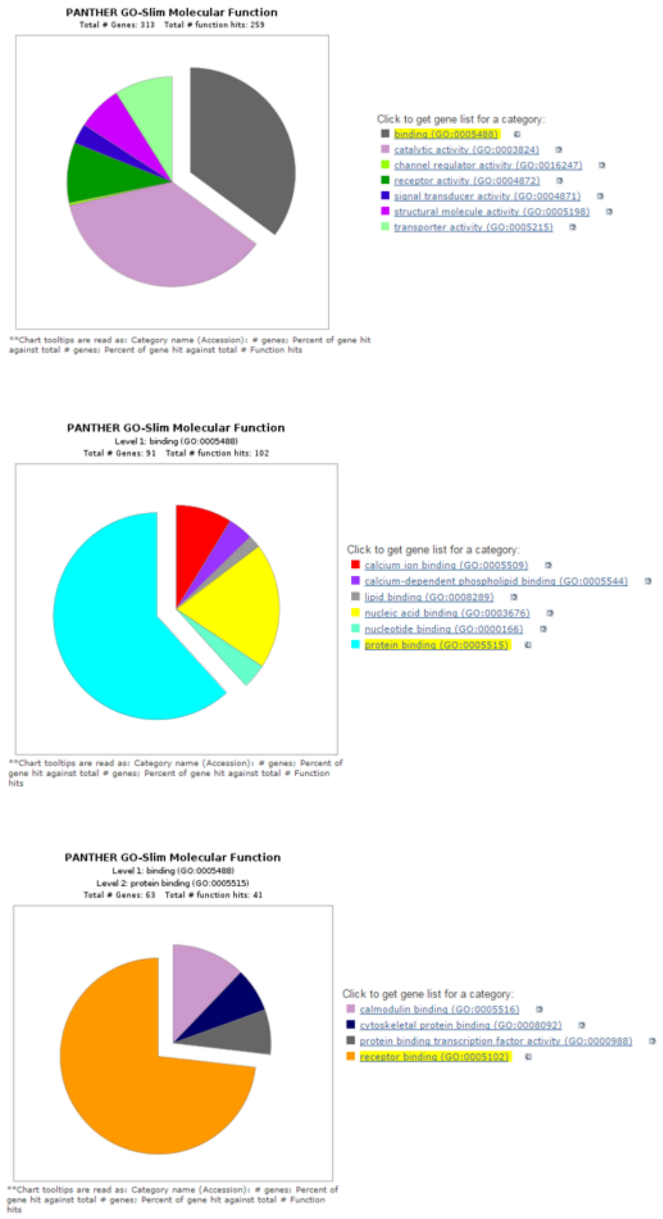

B

HEK293T

(induced in HEK293T$^{PARP1-/-}$ relative to HEK293T^{EV})

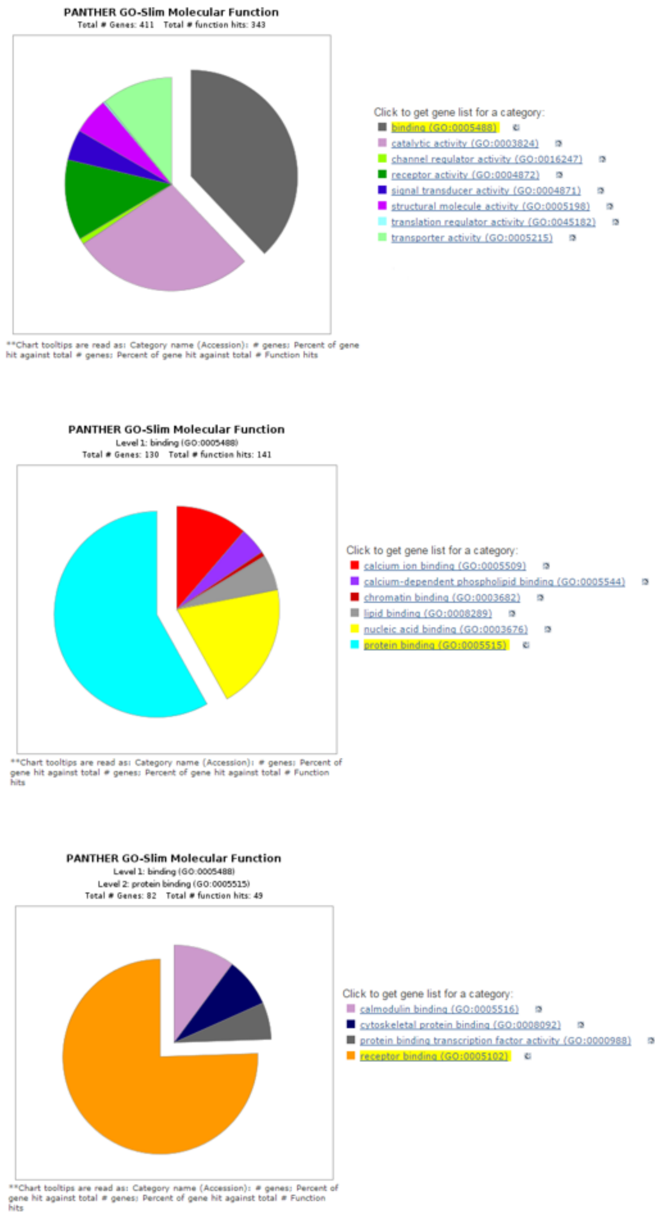

Supplement: S10 Fig — (A) mRNAs induced in HCT116PARP1-/- cells (clones C2 and C4) relative to HCT116EV cells were prominently enriched for Molecular Function: “binding” (level 1); “protein binding” (level 2” and “receptor binding” (level 3). (B) Enrichment for the same hierarchy was observed in HEK293TPARP1-/- cells (clones C5 and C9) relative to HEK293TEV cells. (PDF) [file pone.0194611.s010.pdf]
