# Supplementary material for: PARP1 depletion induces RIG-I-dependent signaling in human cancer cells
Source: PLoS One. 2018 Mar 28;13(3):e0194611. doi: 10.1371/journal.pone.0194611 (PMC5874037; doi:10.1371/journal.pone.0194611)
Supplement: S3 Table — (PDF) [file pone.0194611.s017.pdf]

S3 Table. Gene Set Enrichment Analysis (GSEA; Hallmark) of pathways differentially expressed in HCT116$^{PARP1-/-}$ and HCT-116^{EV} cells

Rank	NAME	SIZE	ES	NES	NOM p-val	FDR q-val	FWER p-val
1	INTERFERON_ALPHA_RESPONSE	97	-0.7231735	-2.1192596	0	0	0
2	INTERFERON_GAMMA_RESPONSE	197	-0.6080619	-1.8955262	0	0	0
3	COMPLEMENT	197	-0.5680321	-1.7659404	0	0.001042424	0.003
4	COAGULATION	134	-0.521067	-1.5714287	0	0.013699746	0.052
5	GLYCOLYSIS	192	-0.49423343	-1.5465838	0	0.017949354	0.084
6	TNFA_SIGNALING_VIA_NFKB	200	-0.49499783	-1.5449282	0.001146789	0.015646571	0.088
7	XENOBIOTIC_METABOLISM	194	-0.49280316	-1.5325973	0.001153403	0.016550716	0.105
8	P53_PATHWAY	191	-0.49366593	-1.5269948	0	0.01593217	0.116
9	IL6_JAK_STAT3_SIGNALING	86	-0.53433394	-1.5118597	0.007633588	0.017360808	0.14
10	APOPTOSIS	158	-0.49061725	-1.5059491	0	0.017277187	0.155
11	PROTEIN_SECRETION	90	-0.5280493	-1.5024619	0.006289308	0.016266033	0.16
12	KRAS_SIGNALING_UP	192	-0.48521218	-1.5013741	0.001141553	0.014986771	0.161
13	TGF_BETA_SIGNALING	52	-0.5702851	-1.4979786	0.008633094	0.014698471	0.171
14	ESTROGEN_RESPONSE_EARLY	193	-0.48061672	-1.4914694	0	0.014675021	0.182
15	ESTROGEN_RESPONSE_LATE	196	-0.47598296	-1.484907	0.003541913	0.015013523	0.198
16	INFLAMMATORY_RESPONSE	195	-0.47079644	-1.4587399	0	0.019632805	0.267
17	PI3K_AKT_MTOR_SIGNALING	99	-0.49217382	-1.4451302	0.013613861	0.021332117	0.301
18	REACTIVE_OXIGEN_SPECIES_PATHWAY	45	-0.5604581	-1.4323187	0.025435073	0.023076735	0.334
19	APICAL_SURFACE	43	-0.5480154	-1.4155499	0.041436464	0.028051723	0.401
20	ALLOGRAFT_REJECTION	192	-0.4466615	-1.3910681	0.013544018	0.036374316	0.505
21	CHOLESTEROL_HOMEOSTASIS	72	-0.4924241	-1.3872652	0.032258064	0.03600661	0.524
22	UV_RESPONSE_DN	142	-0.45878932	-1.3848742	0.018359853	0.035427425	0.533
23	BILE_ACID_METABOLISM	105	-0.47216162	-1.3771594	0.016209476	0.036808584	0.567
24	APICAL_JUNCTION	194	-0.4305168	-1.3313518	0.015046297	0.06063277	0.769
25	MTORC1_SIGNALING	195	-0.42625314	-1.3275641	0.012910798	0.060576834	0.783
26	KRAS_SIGNALING_DN	196	-0.4246284	-1.3176625	0.023809524	0.06424428	0.824
27	EPITHELIAL_MESENCHYMAL_TRANSITION	193	-0.4197962	-1.3013544	0.024475524	0.073584855	0.868
28	PEROXISOME	100	-0.44258863	-1.2929472	0.057971016	0.07763929	0.888
29	ANDROGEN_RESPONSE	98	-0.43698037	-1.2654948	0.08138101	0.09973766	0.946
30	ADIPOGENESIS	195	-0.40235537	-1.254943	0.05359179	0.107063666	0.961
31	MITOTIC_SPINDLE	190	-0.40160128	-1.2507243	0.05232558	0.10781065	0.966
32	OXIDATIVE_PHOSPHORYLATION	188	-0.3780561	-1.1845263	0.12313003	0.19134384	0.998
33	IL2_STAT5_SIGNALING	189	-0.3702865	-1.1528248	0.16132723	0.24174036	0.999
34	FATTY_ACID_METABOLISM	153	-0.38013726	-1.1520032	0.16805722	0.2359132	0.999
35	HYPOXIA	195	-0.35844627	-1.127123	0.2	0.2745556	1
36	UNFOLDED_PROTEIN_RESPONSE	108	-0.3817485	-1.1269919	0.21791768	0.26715347	1
37	ANGIOGENESIS	35	-0.448056	-1.1014447	0.3375	0.31014323	1
38	HEME_METABOLISM	190	-0.352038	-1.0965643	0.26279864	0.31127045	1
39	NOTCH_SIGNALING	32	-0.41864386	-1.0288817	0.4188406	0.44970873	1
40	MYOGENESIS	190	-0.32446653	-1.0123525	0.43463302	0.47639444	1
41	DNA_REPAIR	140	-0.27523893	-0.8306637	0.8469751	0.8785164	1
42	UV_RESPONSE_UP	154	-0.26591897	-0.8066237	0.912154	0.90050495	1
43	PANCREAS_BETA_CELLS	39	-0.31063548	-0.7968479	0.8072626	0.89497006	1
