# Supplementary material for: PARP1 depletion induces RIG-I-dependent signaling in human cancer cells
Source: PLoS One. 2018 Mar 28;13(3):e0194611. doi: 10.1371/journal.pone.0194611 (PMC5874037; doi:10.1371/journal.pone.0194611)
Supplement: S4 Table — (PDF) [file pone.0194611.s018.pdf]

S4 Table. Gene Set Enrichment Analysis (GSEA; Hallmark) of pathways differentially expressed in HEK293T$^{PARP1-/-}$ and HEK293T^{EV} cells

Rank	NAME	SIZE	ES	NES	NOM p-val	FDR q-val	FWER p-val
1	EPITHELIAL_MESENCHYMAL_TRANSITION	193	-0.6078905	-1.808786	0	0	0
2	COAGULATION	134	-0.60683674	-1.7415352	0	4.69E-04	0.001
3	HYPOXIA	195	-0.566417	-1.6724921	0	9.87E-04	0.003
4	INTERFERON_ALPHA_RESPONSE	97	-0.5831463	-1.6296462	0.001101322	0.00220475	0.009
5	INFLAMMATORY_RESPONSE	195	-0.5494204	-1.6190127	0	0.002382589	0.012
6	MYOGENESIS	190	-0.539986	-1.6038028	0	0.00301226	0.017
7	P53_PATHWAY	191	-0.5438985	-1.589939	0	0.003890678	0.025
8	ESTROGEN_RESPONSE_EARLY	193	-0.5336318	-1.5726159	0	0.004995877	0.037
9	APICAL_JUNCTION	194	-0.52978754	-1.5597856	0	0.005415367	0.045
10	ANGIOGENESIS	35	-0.64796686	-1.5597169	0.001257862	0.00487383	0.045
11	INTERFERON_GAMMA_RESPONSE	197	-0.52305853	-1.554975	0	0.004706419	0.048
12	KRAS_SIGNALING_UP	192	-0.52909046	-1.5517617	0	0.004555569	0.051
13	CHOLESTEROL_HOMEOSTASIS	72	-0.5739662	-1.5418502	0.002293578	0.004731371	0.058
14	KRAS_SIGNALING_DN	196	-0.51907325	-1.5339755	0	0.005048287	0.067
15	APOPTOSIS	158	-0.51482	-1.4866298	0.00106383	0.009677648	0.137
16	TNFA_SIGNALING_VIA_NFKB	200	-0.49923053	-1.4730189	0	0.011248629	0.166
17	UV_RESPONSE_DN	142	-0.5076917	-1.4647186	0.002123142	0.01171136	0.181
18	COMPLEMENT	197	-0.49172896	-1.464118	0.001027749	0.011169524	0.182
19	APICAL_SURFACE	43	-0.57361156	-1.4601028	0.021951219	0.011108418	0.191
20	XENOBIOTIC_METABOLISM	194	-0.4909275	-1.4436557	0.001033058	0.013459718	0.237
21	ESTROGEN_RESPONSE_LATE	196	-0.48280722	-1.4257455	0.002068252	0.01694151	0.301
22	ANDROGEN_RESPONSE	98	-0.4955746	-1.3829273	0.011025358	0.030050064	0.482
23	MTORC1_SIGNALING	195	-0.4698128	-1.3812995	0.002089864	0.029818155	0.492
24	NOTCH_SIGNALING	32	-0.5678233	-1.3783054	0.060606062	0.029950138	0.508
25	IL2_STAT5_SIGNALING	189	-0.46318975	-1.3773882	0.005144033	0.028986499	0.511
26	HEME_METABOLISM	190	-0.46437502	-1.3736418	0.006179197	0.029135182	0.532
27	BILE_ACID_METABOLISM	105	-0.473889	-1.3456111	0.026402641	0.039649837	0.658
28	ADIPOGENESIS	195	-0.4509695	-1.3365153	0.006179197	0.042865966	0.697
29	TGF_BETA_SIGNALING	52	-0.51868993	-1.3292093	0.06642942	0.045274843	0.725
30	UV_RESPONSE_UP	154	-0.452459	-1.3090008	0.020212766	0.05731284	0.833
31	GLYCOLYSIS	192	-0.43759042	-1.2988298	0.037228543	0.06319348	0.86
32	ALLOGRAFT_REJECTION	192	-0.43206972	-1.2812115	0.024896266	0.07566405	0.92
33	IL6_JAK_STAT3_SIGNALING	86	-0.466928	-1.2760984	0.06476399	0.078101754	0.927
34	PROTEIN_SECRETION	90	-0.45339867	-1.2543479	0.087100334	0.09622343	0.961
35	SPERMATOGENESIS	131	-0.43167856	-1.2494609	0.07675906	0.098712765	0.968
36	HEDGEHOG_SIGNALING	35	-0.507874	-1.2452228	0.16020025	0.09971885	0.97
37	REACTIVE_OXIGEN_SPECIES_PATHWAY	45	-0.45985314	-1.1621492	0.23459716	0.21264891	1
38	PANCREAS_BETA_CELLS	39	-0.4654403	-1.1553684	0.24659231	0.21925613	1
39	FATTY_ACID_METABOLISM	153	-0.3772672	-1.1046191	0.24683544	0.3145066	1
40	PEROXISOME	100	-0.38680843	-1.0683011	0.36013216	0.390871	1
41	WNT_BETA_CATENIN_SIGNALING	41	-0.4174919	-1.0391965	0.41970804	0.4510481	1
42	UNFOLDED_PROTEIN_RESPONSE	108	-0.36153677	-1.0120093	0.4728261	0.50755036	1
43	MITOTIC_SPINDLE	190	-0.33571637	-0.99184453	0.5190525	0.54357266	1
44	DNA_REPAIR	140	-0.29608893	-0.85394716	0.8065539	0.8301927	1
45	OXIDATIVE_PHOSPHORYLATION	188	-0.27976057	-0.8287381	0.8695198	0.8549417	1
46	PI3K_AKT_MTOR_SIGNALING	99	-0.28228265	-0.7886767	0.8698031	0.8929346	1
