# Supplementary material for: PARP1 depletion induces RIG-I-dependent signaling in human cancer cells
Source: PLoS One. 2018 Mar 28;13(3):e0194611. doi: 10.1371/journal.pone.0194611 (PMC5874037; doi:10.1371/journal.pone.0194611)
Supplement: S5 Table — (PDF) [file pone.0194611.s019.pdf]

S5 Table. Canonical pathways differentially regulated in HCT116$^{PARP1-/-}$ cells relative to HCT116^{EV} cells (IPA)

**Canonical pathway**	**Genes**
Atherosclerosis Signaling	*ALB*, *ALOX12*, *APOL1*, *CCR3*, *CD36*, *IL8*, *IL18*, *IL1A*, *IL1RN*, *IL36G*, *IL36RN*, *ITGA4*, *ITGB2*, *LYZ*, *MMP1*, *MMP13*, *ORM2*, *PDGFB*, *PLA2G4D*, *PLA2G4E*, *PLB1*, *SELPLG*, *SERPINA1*
Complement System	*C3*, *CD59*, *CFB*, *CFH*, *CFI*, *ITGAX*, *ITGB2*, *MASP1*
Coagulation System	*BDKRB1*, *BDKRB2*, *FGG*, *KLKB1*, *PLAU*, *SERPINA1*, *SERPINA5*, *SERPINF2*
Granulocyte Adhesion & Diapedesis	*CCL20*, *CCL26*, *CLDN1*, *CLDN4*, *CLDN9*, *CXCL1*, *CXCL3*, *IL8*, *IL18*, *IL1A*, *IL1R2*, *IL1RN*, *IL36G*, *IL36RN*, *ITGA3*, *ITGA4*, *ITGB1*, *ITGB2*, *MMP1*, *MMP13*, *SDC4*, *SELL*, *SELPLG*
Agranulocyte Adhesion & Diapedesis	*ACTA2*, *ACTG2*, *CCL20*, *CCL26*, *CLDN1*, *CLDN4*, *CLDN9*, *CXCL1*, *CXCL3*, *IL8*, *CXCR1*, *FN1*, *IL18*, *IL1A*, *IL1RN*, *IL36G*, *ITGA3*, *ITGA4*, *ITGB1*, *ITGB2*, *ITGB7*, *MMP1*, *MMP13*, *MMP28*, *SDC4*, *SELL*, *SELPLG*
Communication between Innate and Adaptive Immune Cells	*CCR7*, *CSF2*, *IL8*, *IL18*, *IL1A*, *IL1RN*, *IL36G*, *TLR3*, *TLR7*, *TLR10*, *TNFSF13B*
Role of Pattern Recognition Receptors in Recognition of Bacteria and Viruses	*C3*, *CLEC7A*, *CSF2*, *IL8*, *DDX58*, *IFIH1*, *IL18*, *IL1A*, *IRF7*, *LIF*, *OAS1*, *OAS2*, *OSM*, *SYK*, *TLR3*, *TLR7*
Hepatic Stellate Cell Activation	*ACTA2*, *CCR7*, *COL16A1*, *COL17A1*, *COL24A1*, *COL27A1*, *COL4A5*, *COL5A1*, *COL6A1*, *COL9A3*, *CXCL3*, *IL8*, *CXCR3*, *EDN1*, *EGF*, *FAS*, *FLT1*, *FLT4*, *FN1*, *IGFBP5*, *IL1A*, *IL1R2*, *LAMA1*, *MET*, *MMP1*, *MMP13*, *PDGFB*, *TGFA*
NF-κB signaling	*AKT3*, *BMPR1B*, *CARD11*, *EGF*, *FLT1*, *FLT4*, *IL18*, *IL1A*, *IL1R2*, *IL1RN*, *IL36G*, *IL36RN*, *NTRK2*, *TANK*, *TGFA*, *TLR3*, *TLR7*, *TLR10*, *TNFSF13B*
LXR/RXR Activation	*ALB*, *APOL1*, *C3*, *CD36*, *IL18*, *IL1A*, *IL1R2*, *IL1RN*, *IL36G*, *IL36RN*, *LYZ*, *NOS2*, ORM2, *PON3*, *SERPINA1*, *SERPINF2*, *TLR3*
Acute Phase Response Signaling	*AKT3*, *ALB*, *C3*, *CFB*, *FGG*, *FN1*, *HRG*, *IL18*, *IL1A*, *IL1RN*, *IL36G*, *IL36RN*, *ITIH2*, *KLKB1*, *NR3C1*, *ORM2*, *OSM*, *RBP3*, *SERPINA1*, *SERPINA3*, *SERPINF2*
FXR/RXR Activation	*AKT3*, *ALB*, *APOL1*, *C3*, *CYP19A1*, *FBP1*, *FGF19*, *IL18*, *IL1A*, *IL1RN*, *IL36G*, *IL36RN*, *ORM2*, *PON3*, *SERPINA1*, *SERPIF2*, *SLCO1B1*
Role of Macrophages, Fibroblasts and Endothelial Cells in Rheumatoid Arthritis	*ADAMTS4*, *AKT3*, *CAMK4*, *CAMK2B*, *CCND1*, *CSF2*, *CXCL8*, *FN1*, *IL7*, *IL18*, *IL1A*, *ILR2*, *IL1RN*, *IL36G*, *IL36RN*, *MMP1*, *MMP13*, *NOS2*, *OSM*, *PDGFB*, *PPP3R2*, *TLR3*, *TLR7*, *TLR10*, *TNFSF13B*, *TRAF4*, *WNT11*, *WNT7A*, *WNT7B*, *WNT9A*
LPS/IL-1 Mediated Inhibition of RXR Function	*ABCC3*, *ALDH1A3*, *ALDH3A1*, *ALDH3B2*, *CHST4*, *CHST10*, *CHST13*, *CYP3A7*, *FMO1*, *FMO5*, *GSTA1*, *GSTA2*, *IL18*, *IL1A*, *IL1R2*, *IL1RN*, *IL36G*, *IL36RN*, *MGST2*, *NDST3*, *PAPSS2*, *SLCO1A2*, *SULT1E1*
Crosstalk between Dendritic Cells and Natural Killer Cells	*ACTA2*, *ACTG2*, *CAMK2B*, *CCR7*, *CSF2*, *FAS*, *FSCN2*, *IL18*, *IL2RB*, *TLR3*, *TLR7*, *TNFSF10*
Role of Osteoblasts, Osteoclasts and Chondrocytes in Rheumatoid Arthritis	*ADAMTS4*, *AKT3*, *BIRC3*, *CAMK4*, *CSF2*, *IL7*, *IL18*, *IL1A*, *IL1R2*, *IL1RN*, *IL36G*, *IL36RN*, *ITGA3*, *ITGB1*, *MMP1*, *MMP13*, *PPP3R2*, *RUNX2*, *SMAD6*, *WNT11*, *WNT7A*, *WNT7B*, *WNT9A*
TREM1 Signaling	*AKT3*, *CIITA*, *CSF2*, *CXCL3*, *CXCL8*, *IL18*, *ITGAX*, *ITGB1*, *NLRP6*, *STAT5A*, *TL3*, *TLR7*, *TLR10*
Caveolar-mediated Endocytosis Signaling	*ACTA2*, *ACTG2*, *ALB*, *EGF*, *ITGA3*, *ITGA4*, *ITGA9*, *ITGAX*, *ITGB1*, *ITGB2*, *ITGB4*, *ITGB6*, *ITGB7*
Altered T and B Cell Signaling in Rheumathoid Arthritis	*CSF2*, *FAS*, *HLA-DOB*, *IL18*, *IL1A*, *IL1RN*, *IL23A*, *IL36G*, *IL36RN*, *TLR3*, *TLR7*, *TLR10*, *TNFSF13B*
VDR/RXR Activation	*CDKN1A*, *CSF2*, *IGFBP5*, *IGFBP6*, *KLF4*, *KLK6*, *RUNX2*, *SEMA3B*, *SERPINB1*, *TRPV6*
PI3K Signaling in B Lymphocytes	*AKT3*, *BLNK*, *C3*, *CAMK4*, *CAMK2B*, *DAPP1*, *INPP5D*, *IRS4*, *PIK3AP1*, *PLEKHA2*, *PPP3R2*, *SYK*
