# Supplementary material for: PARP1 depletion induces RIG-I-dependent signaling in human cancer cells
Source: PLoS One. 2018 Mar 28;13(3):e0194611. doi: 10.1371/journal.pone.0194611 (PMC5874037; doi:10.1371/journal.pone.0194611)
Supplement: S6 Table — (PDF) [file pone.0194611.s020.pdf]

S6 Table. Canonical pathways differentially regulated in HEK293T$^{PARP1-/-}$ cells relative to HEK293^{EV} cells (IPA)

**Canonical pathway**	**Genes**
Antigen Presentation	*HLA-DMA*, *HA-DOA*, *HLA-DPA1*, *HLA-DPB1*, *HLA-DQA1*, *HLA-DRA*, *PSMB8*, *PSMB9*
B cell Development	*CD19*, *CD79B*, *HLA-DMA*, *HLA-DOA*, *HLA-DQA1*, *HAL-DQB1*, *HLA-DRA*, *SPN*
T cell Helper Differentiation	*HLA-DMA*, *HLA-DOA*, *HLA-DQA1*, *HLA-DQB1*, *HLA-DRA*, *IL17F*, *IL21R*, *IL6R*, *NGFR*, *TBX21*, *TGFB1*, *TNFRSF11B*, *TNFRSF1B*
Complement System	*C3*, *C1R*, *C5AR1*, *CFD*, *CFI*, *CR2*, *ITGB2*, *MASP1*, *SERPING1*
Coagulation System	*A2M*, *BDKRB1*, *BDKRB2*, *F10*, *F12*, *FGB*, *PLAT*, *PLAU*, *SERPINC1*, *SERPINE1*, *SERPINF2*, *TFPI*, *THBD*, *VWF*
Granulocyte Adhesion and Diapedesis	*C5AR1*, *CCL2*, *CCL26*, *CLDN1*, *CLDN6*, *CLDN7*, *CLDN23*, *CXCL6*, *CXCL8*, *CXCL16*, *CXCL17*, *HRH1*, *HRH4*, *HSPB1*, *IL1R2*, *ITGA5*, *ITGB2*, *ITGB3*, *MMP2*, *MMP11*, *MMP12*, *MMP13*, *MMP19*, *MMP23B*, *NGFR*, *PF4*, *SDC1*, *SELL*, *SELPLG*, *TNFRSF11B*, *TNFRSF1B*
PI3K Signaling in B Lymphocytes	*ATF3*, *BLK*, *BLNK*, *BTK*, *C3*, *CAMK2B*, *CD19*, *CD79B*, *CR2*, *CAPP1*, *INPP5D*, *NOTUM*, *PI3KAP1*, *PLEKHA2*, *PLEKHA4*, *PRKCB*, *RRAS*, *SYK*
Hepatic Stellate Activation	*A2M*, *ACTA2*, *AGT*, *CCL2*, *CCR7*, *COL11A1*, *COL12A1, COL15A1*, *COL16A1*, *COL20A1*, *COL21A1*, *COL3A1*, *COL4A3*, *COL4A4*, *COL5A3*, *COL6A2*, *COL6A3*, *COL6A5*, *COL6A6*, *COL8A1*, *COL9A3*, *CSCL8*, *EDNRA*, *EDNRB*, *EGF*, *FN1*, *HGF*, *IGFBP3*, *IGFBP4*, *IL1R2*, *IL6R*, *MMP2*, *MMP13*, *MYL1*, *NGFR*, *PDGFB*, *PDGFD*, *SERPINE1*, *TGFB1*, *TIMP2*, *TNFRSF11B*, *TNFRSF1B*
Atherosclerosis Signaling	*ALOX5*, *ALOX15*, *ALOX15B*, *ALOXE3*, *APOA1*, *APOD*, *APOE*, *CCL2*, *CD36*, *CLU*, *COL3A1*, *COL5A3*, *CXCL8*, *ITGB2*, *LPL*, *MMP13*, *MSR1*, *PDGFB*, *PDGFD*, *PLA2G3*, *PLA2G16*, *PLA2G4C*, *SELPLG*, *TGFB1*, *TNFRSF12A*
FXR/RXR Activation	*ABCB4*, *ABCG5*, *AGT*, *AHSG*, *APOA1*, *APOD*, *APOE*, *C3*, *CLU*, *CYP19A1*, *FABP6*, *FGF19*, *FOXA2*, *FOXA3*, *LPL*, *MTTP*, *PCK2*, *PPARGC1A*, *SDC1*, *SERPINF1*, *SERPINF2*, *SULT2A1*, *VLDLR*
Agranulocyte Adhesion and Diapedesis	*ACTA2*, *C5AR1*, *CCL2*, *CCL26*, *CD34*, *CLDN1*, *CLDN6*, *CLDN7*, *CLDN23*, *CXCL6*, *CXCL8*, *CXCL16*, *CXCL17*, *FN1*, *HRH1*, *ITGA5*, *ITGB2*, *MADCAM1*, *MMP2*, *MMP11*, *MMP12*, *MMP13*, *MMP19*, *MMP23B*, *MYL1*, *PF4*, *SELL*, *SELPLG*
LPS/IL-1 Mediated Inhibition of RXR Function	*ABCA1*, *ABCG5*, *ACSBG1*, *ALDH1A1*, *ALDH1A3*, *APOE*, *CHST1*, *CHST4*, *CHST5*, *CHST13*, *CYP2C8*, *CYP3A4*, *FABP6*, *FMO3*, *FMO4*, *GSTO2*, *HS3ST5*, *HS3ST6*, *IL1R2*, *MAOA*, *MAOB*, *MGST1*, *NGFR*, *PPARGC1A*, *SLC27A6*, *SULT1C4*, *SULT2A1*, *SULT2B1*, *TNFRSF11B*, *TNFRSF1B*
Role of Macrophages, Fibroblasts and Endothelial Cells in Rheumatoid Arthritis	*ADAMTS4*, *C5AR1*, *CAMK2B*, *CCL2*, *CCND1*, *CXCL8*, *DKK1*, *DKK3*, *DKKL1*, *FN1*, *FRZB*, *IL32*, *IL17RC*, *IL1R2*, *IL6R*, *LRP1*, *LTB*, *MMP13*, *NGFR*, *NOTUM*, *OSM*, *PDGFB*, *PDGFD*, *PIK3R5*, *PRKCB*, *PRKCH*, *ROR2*, *RRAS*, SFRP4, *SFRP5*, *SOCS3*, *TGFB1*, *TNFRSF11B*, *TNFRSF1B*, *WIF1*, *WNT2*, *WNT6*, *WNT11*, *WNT3A*
LXR/RXR Activation	*ABCA1*, *ABCG5*, *AGT*, *AHSG*, *APOA1*, *APOD*, *APOE*, *C3*, *CCL2*, *CD36*, *CLU*, *IL1R2*, *LPL*, *MSR1*, *NGFR*, *SERPINF1*, *SERPINF2*, *TNFRSF11B*, *TNFRSF1B*
Role of Osteoblasts, Osteoclasts and Chondrocytes in Rheumatoid Arthritis	*ADAMTS4*, *ADAMTS5*, *ALPL*, *BIRC3*, *BMP5*, *CALCR*, *DKK1*, *DKK3*, *DKKL1*, *FRZB*, *GSN*, *IL11*, *IL1R2*, *ITGA5*, *ITGB3*, *LRP1*, *MMP13*, *NGFR*, *PIK3R5, SFRP4*, *SFRP5*, *SPP1*, *TGFB1*, *TNFRSF11B*, *TNFRSF1B*, *WIF1*, *WNT2*, *WNT6*, *WNT11*, *WNT3A*
Acute Phase Response Signaling	*A2M*, *AGT*, *AHSG*, *APOA1*, *C3*, *C1R*, *CRABP2*, *FGB*, *FN1*, *IL6R*, *NGFR*, *OSM*, *RRAS*, *SERPINE1*, *SERPINF1*, *SERPINF2*, *SERPING1*, *SOCS3*, *TNFRSF11B*, *TNFRSF1B*, *VWF*
VDR/RXR Activation	*CALB1*, *CDKN1A*, *CYP24A1*, *GADD45A*, *IGFBP3*, *IGFBP6*, *PRKCB*, *PRKCH*, *SPP1*, *SULT2A1*, *THBD*
