# Supplementary material for: PARP1 depletion induces RIG-I-dependent signaling in human cancer cells
Source: PLoS One. 2018 Mar 28;13(3):e0194611. doi: 10.1371/journal.pone.0194611 (PMC5874037; doi:10.1371/journal.pone.0194611)
Supplement: S7 Table — (PDF) [file pone.0194611.s021.pdf]

S7 Table. List of primers used in the study and literature references if applicable

Primer ID	Sequence
LY6D_Fwd	AACTGCAAGCATTCTGTGGTCTG
LY6D_Rev	ACTCCGCACAGTCCTTCTTCAC
IGFBP5_Fwd	AGCAGTGAAGAAGGACCGCA
IGFBP5_Rev	CAGGGAAGCCTCCATGTGTCT
SLC5A1_Fwd	GCTGCATCCTGTGTGGGTATCT
SLC5A1_Rev	AAGGTTGGATAGGCGATGTTGGTA
KLK5_Fwd	CGCTGTTGCTAAGGCCCAAC
KLK5_Rev	TGACCCCCTGGAACATCTGC
OAS1_Fwd	GAGTTCGATGTGCTGCCTGC
OAS1_Rev	CCCTCTTTCTGCAGGTCGGT
ZNF804A_Fwd	GGAAAAGGCACTCCAACGCC
ZNF804A_Rev	ACAGTTGTTGATTTGAACATGGGGC
HOXB8_Fwd	CCACACAGCTCTTCCCCTGG
HOXB8_Rev	TCGATTCGCCGCTTACGAGT
TM4SF18_Fwd	ACTGCCTGGTCATCTCTGCC
TM4SF18_Rev	CCACAACATGTGCAGGTTCCA
PODXL2_Fwd	GCCCCTGGAGACATGGAACT
PODXL2_Rev	AGTTTTTCCCAGCCAGATTGCTC
LRRC7_Fwd	TGGCTACCTTCATAGTCTTCGGAC
LRRC7_Rev	TGCATCTGTCCAATCTCTTCAGGA
PGR_Fwd	CGTCAGTGGGCAGATGCTGT
PGR_Rev	TGTGGGATCTGCCACATGGT
LGR6_Fwd	TGGACTTGGGGGCTTGATGC
LGR6_Rev	CACTGCCCAGAGGCCTTGAA
PTN_Fwd	CCCCTGCAACTGGAAGAAGC
PTN_Rev	GCAGGGCTCGCTTCAGACTT
KCTD8_Fwd	AACCAGTACCGCGACGACAA
KCTD8_Rev	AGCTCATTACAGGAAGTCCCACT
CTSZ_Fwd	TGGAGGGTGGGAGACTACGG
CTSZ_Rev	ATGCCTCCGGTGTAGTTAGCC
OSMR_Fwd	CTTGGGATCCTGGGACGGAC
OSMR_Rev	GCCAATTACACCAGTTTTTGTGTGT
ZIK1_Fwd	TCACAGGACGAGTGGGGACT
ZIK1_Rev	TTCTGTGTGGATGAACCTGCCT
STK31_Fwd	TGAAGATCAGTGTTGGTACAGATGC
STK31_Rev	ACTGCAGCTCCAAAGGAATTTCA
APOBEC3G_Fwd	GTGGAGCGCATGCACAATGA
APOBEC3G_Rev	GGTCCAGGTCCAGCTTCCAA
APOBEC3H_Fwd	TTAACAACAAGCGCCGCCTC
APOBEC3H_Rev	TGCGTTTCGTCCAGTCCCAT

Primer ID	Sequence
APOBEC3C_Fwd	GCCAACGATCGGAACGAAACT
APOBEC3C_Rev	AGGAGACAGTATGTCGTCGCAG
APOBEC3D_Fwd	GGCCCGGTACTACCCAAACG
APOBEC3D_Rev	GCAGGCAGGGGTTCCATGAT
APOBEC3F_Fwd	TCCCTCAAGGCCCCGTTTG
APOBEC3F_Rev	ACTTGTAAGCAGGCAGCTGGT
IRF9_Fwd	ACCATCAAAGCGACAGCACAG
IRF9_Rev	TGTCTGAATGGACTGCTCCCC
MX1_Fwd	GGCAAGGTCAGTTACCAGGACT
MX1_Rev	GCCACTCTGGTTATGCCAGGA
MYD88_Fwd	ACCTGCAGAGCAAGGAATGTGA
MYD88_Rev	CTTGGCAAGGCGAGTCCAGA
IFI27_Fwd	CGCCTCGTCCTCCATAGCAG
IFI27_Rev	ATGGCAGACCCAATGGAGCC
STING_Fwd	CCATGGGCTGGCATGGTCATA
STING_Rev	AGGCACCCCACAGTCCAATG
IFI6_Fwd	GCTGATGAGCTGGTCTGCGA
IFI6_Rev	GTAGCCCATCAGGGCACCAA
IFIH1_Fwd	CTTCACCTGGTGTTGGAGGGG
IFIH1_Rev	GCAAACTTCTTGCATGGCTCCT
RIG-I_Fwd	ACGCCTTCAGACATGGGACG
RIG-I_Rev	ACGTCAGCTGTGTAACATGCCA
IFI16_Fwd	AGAAGTGCCAGCGTAACTCCTAA
IFI16_Rev	GTCGTCCATGCACCACCACT
IFIT1_Fwd	GGCTGCCTAATTTACAGCAACCA
IFIT1_Rev	TGCTCCAGACTATCCTTGACCTGA
IFIT2_Fwd	CCCTGCCGAACAGCTGAGAA
IFIT2_Rev	AGTTGCCGTAGGCTGCTCTC
IFITM1_Fwd	TTGAACTGGTGCTGTCTGGGC
IFITM1_Rev	TGGTAGACTGTCACAGAGCCGAA
IRF7_Fwd	AGGCAGAGCCGTACCTGTCA
IRF7_Rev	ATAGGAACGTGCAGCTCGGG
RelA_Fwd	GCTCTGCTTCCAGGTGACAGT
RelA_Rev	CAGAGTTTCGGTTCACTCGGC
G1P2_Fwd	AGTACAGGAGCTTGTGCCGTG
G1P2_Rev	CTTCTGGGTGATCTGCGCCT
STAT1_Fwd	AGCAAGTTCGGCAGCAGCTTA
STAT1_Rev	GCATGCAGGGCTGTCTTTCC
cGAS_Fwd	CCAACACTCGTGCATATTACTTTGT
cGAS_Rev	AGCAGGGCTCCCTCCTCTTT

Primer ID	Seq.			
TBK1_Fwd	AGGCCAAGGAGCTACTGCAAAT		IFNA14_Rev	TCCTGTTATTCAGGCTGTGGGTT
TBK1_Rev	AACATCCACTGGACGAAGGAAGC		IFI27L2_Fwd	TCACTGGGGCAGGAATCGC
IRF3_Fwd	GGGACTTTTCCCAGCCAGACA		IFI27L2_Rev	AGGTGAATTCCCCAAGCAGGC
IRF3_Rev	GTGGGGCCAACACCATGTTAC		IFI27L1_Fwd	CCTCAGTAGGAATCGCCGCA
TLR3_Fwd	TGCCTTCTGCACGAATTTGACTG		IFI27L1_Rev	CCCAAGAGCTGTCCCAGCAAA
TLR3_Rev	TTTCCAGCTGAACCTGAGTTCCT		IFNA6_Fwd	ACCTCTTCAGCACAAAGGACTCA
TLR7_Fwd	TGATCTTGGCACCTCTCATGCT		IFNA6_Rev	ACCTCCTGCATCACACAGGC
TLR7_Rev	CCAGAGTGACATCACAGGGCA		IFNA4/7/10_Fwd	CCCAGAGGAGGAGTTTGATGGC
TLR9_Fwd	AAGGGACCTCGAGTGTGAAGC		IFNA4/7/10_Rev	AGGCTCTGTTCCCAAGCAGC
TLR9_Rev	TGATGGCCTGCACCAGGAGA		IFNL1_Fwd	GCAGCTCTCCTGTCTTCCCC
IFI44_Fwd	TCTGTGTGACTCACTGGGGC		IFNL1_Rev	CAGGACCTTCAGCGTCAGGG
IFI44_Rev	TGTCCTTCAGCGATGGGGAAT		MAVS_Fwd	AGCAGGACACAGAACTGGGC
IFI44L_Fwd	CATGTGACTGGCCAAGCCGTA		MAVS_Rev	AGCCAGGGGATGAGGAGGAG
IFI44L_Rev	TGCCCCATCTAGCCCCATAGT		p21_Fwd	TTGTACCCTTGTGCCTCGCT
STAT2_Fwd	TGACCACGGGTTGGAACAGC		p21_Rev	TCCTCTTGGAGAAGATCAGCCG
STAT2_Rev	AACTCTGTGACCTGGGCGTT			
IFNB1_Fwd	AGGACGCCGCATTGACCATC			
IFNB1_Rev	CTGCTCATGAGTTTTCCCCTGGT			
IFNA1/13_Fwd	GCCTGGGAGGTTGTCAGAGC			
IFNA1/13_Rev	GCATGGTCATAGTTATAGCAGGGGT		IFIT3_Fwd [1]	CAGAACTGCAGGGAAACAGC
IFNA14_Fwd	CATCTTCGGGATTCCCAATGGC		IFIT3_Rev[1]	TGAATAAGTTCCAGGTGAAATGGC

References:

1. Cheon H, *et al.* (2013) IFNbeta-dependent increases in STAT1, STAT2, and IRF9 mediate resistance to viruses and DNA damage. *EMBO J* 32(20):2751-2763.
